# Differentiated surface fungal communities at point of harvest on apple fruits from rural and peri-urban orchards

**DOI:** 10.1038/s41598-017-17436-5

**Published:** 2018-02-01

**Authors:** Youming Shen, Jiyun Nie, Zhixia Li, Haifei Li, Yonglong Wu, Yafeng Dong, Jianyi Zhang

**Affiliations:** 1 Institute of Pomology, Chinese Academy of Agricultural Sciences, Xingcheng, 125100 P.R. China; 2 Laboratory of Quality & Safety Risk Assessment for Fruit (Xingcheng), Ministry of Agriculture, Xingcheng, 125100 P.R. China; 3 Quality Inspection and Test Center for Fruit and Nursery Stocks (Xingcheng), Ministry of Agriculture, Xingcheng, 125100 P.R. China

## Abstract

The diverse fungal communities that colonize fruit surfaces are closely associated with fruit development, preservation and quality control. However, the overall fungi adhering to the fruit surface and the inference of environmental factors are still unknown. Here, we characterized the fungal signatures on apple surfaces by sequencing internal transcribed spacer 1 (ITS1) region. We collected the surface fungal communities from apple fruits cultivated in rural and peri-urban orchards. A total of 111 fungal genera belonging to 4 phyla were identified, showing remarkable fungal diversity on the apple surface. Comparative analysis of rural samples harboured higher fungal diversity than those from peri-urban orchards. In addition, fungal composition varied significantly across apple samples. At the genus level, the protective genera *Coniothyrium*, *Paraphaeosphaeria* and *Periconia* were enriched in rural samples. The pathogenic genera *Acremonium*, *Aspergillus*, *Penicillium* and *Tilletiposis* were enriched in peri-urban samples. Our findings indicate that rural samples maintained more diverse fungal communities on apple surfaces, whereas peri-urban-planted apple carried potential pathogenic risks. This study sheds light on ways to improve fruit cultivation and disease prevention practices.

## Introduction

Apples are an important temperate tree fruits worldwide^1,2^. Apple surfaces teem with a wide variety of microorganisms, mainly fungi, that are closely associated with fruit development, post-harvest preservation and quality control^3–5^. Specifically, certain fungi may be recognized as endophytes and bio-control agents, providing a competitive advantage over biotic and abiotic stresses^6^. Phytopathogenic fungi are able to cause plant disease or lead to fruit post-harvest deterioration, significantly affecting fruit yield, quality and marketing value^7,8^. In addition, fungi can produce toxic secondary metabolites, namely, mycotoxins^9^. The accumulation of mycotoxins such as patulin, alternariol toxins, ochratoxins and aflatoxins in fruits and the derived products can seriously affect quality and cause consumer concerns^9,10^. Despite the improvement of technologies for disease control and post-harvest preservation, the complexities of fungal communities and their diversity on apple surfaces, as well as their potential effect on quality, are just beginning to be revealed.

Previous work has characterized fungi in fruit tissues of tens to a hundred species using traditional culture-based techniques^11–13^. Culture-based techniques play important roles in research related to the diagnosis of fungal infections, the confirmation of pathogenicity, and clarification of certain invasive biological mechanisms^14,15^. However, only a small proportion (<5%) of microbes are cultivable^16^, which prevents researchers from understanding the overall fungal community. In recent years, next-generation high-throughput DNA sequencing techniques with improved sequencing capability have been highlighted in microbial community analyses^17–20^. Fungal ribosomal DNA (rDNA) internal transcribed spacer sequences (ITS) are regarded as the credible regions for fungal identification at the species level^21^. Next-generation sequencing platforms (including SOLiD, Illumina and 454 sequencing) coupled with a powerful database and user-friendly software are beneficial for the analysis of a complex microbial community at deeper, more comprehensive level^17,22^. To date, next-generation high-throughput DNA sequencing techniques have been successfully applied in microbial community analyses of various environmental and organic samples, such as soil^23^, air^18^, water^24^, gut^20,25^, leaves and roots^26^. Over the past decade, several studies of fruit microbial communities have been conducted using next-generation sequencing methods^27–29^. However, only a few studies have reported the fungal diversity of apple fruit. Glenn *et al*. first studied the microbial diversity of apple leaves and fruit using a sequencing technique, but the samples were subject to cryopreservation over an extended period^30^. Abdelfattah *et al*. recently reported the fungal communities of organically and conventionally grown apples at the consumer point of purchase using ITS1 sequencing^31^. However, fungi on apple surfaces are still not well known. Furthermore, the effects of environmental factors associated with rural and peri-urban planting on the apple-surface fungi must be further explored.

We expected the fungal community on apple surfaces to be determined by various factors. Fruit species^28^, location^32^, orchard production strategies^19^, and organic/conventional agricultural practices^32^ have been investigated and demonstrated to be important factors that affect microbial community and diversity. Based on FAO statistics (www.faostat.fao.org) for 2014, China is the largest producer of apples and devotes the largest harvest area to apples worldwide, accounting for 48.4% (4.09 × 10^7^/8.46 × 10^7^ tonnes) and 45.0% (2.27 × 10^6^/5.05 × 10^6^ hectare) respectively. In China, many apple trees are planted on hilly land because of ancient planting habits and land-use policy^33^. The Bohai Bay area and Northwest Loess Plateau region— largely hilly, rural areas— are mainly Chinese apple-producing areas (accounting for approximately 80% of the total acreage)^34^. In recent decades, many peri-urban areas have become sightseeing and fruit-picking parks, as part of efforts to explore tourism resources^35,36^. Many apple orchards have recently been planted near cities to attract citizens to fruit-picking activities^37^. Driven by economic interests, the shift in apple fruit cultivation areas from rural to peri-urban has recently become imperative. However, a variety of urban pollutants such as organic toxins, harmful gases, heavy metals, and pathogenic microorganisms are contaminating water, soil, and air near the city^38,39^. Several studies have demonstrated that heavy metals and organic toxins in peri-urban areas can ultimately contaminate agricultural products grown in these regions^40,41^. However, no reference exists to compare the microbial differences among agricultural produce grown in peri-urban and traditional planting areas. The microbial security of peri-urban planted produce requires further attention.

**Figure 1:**
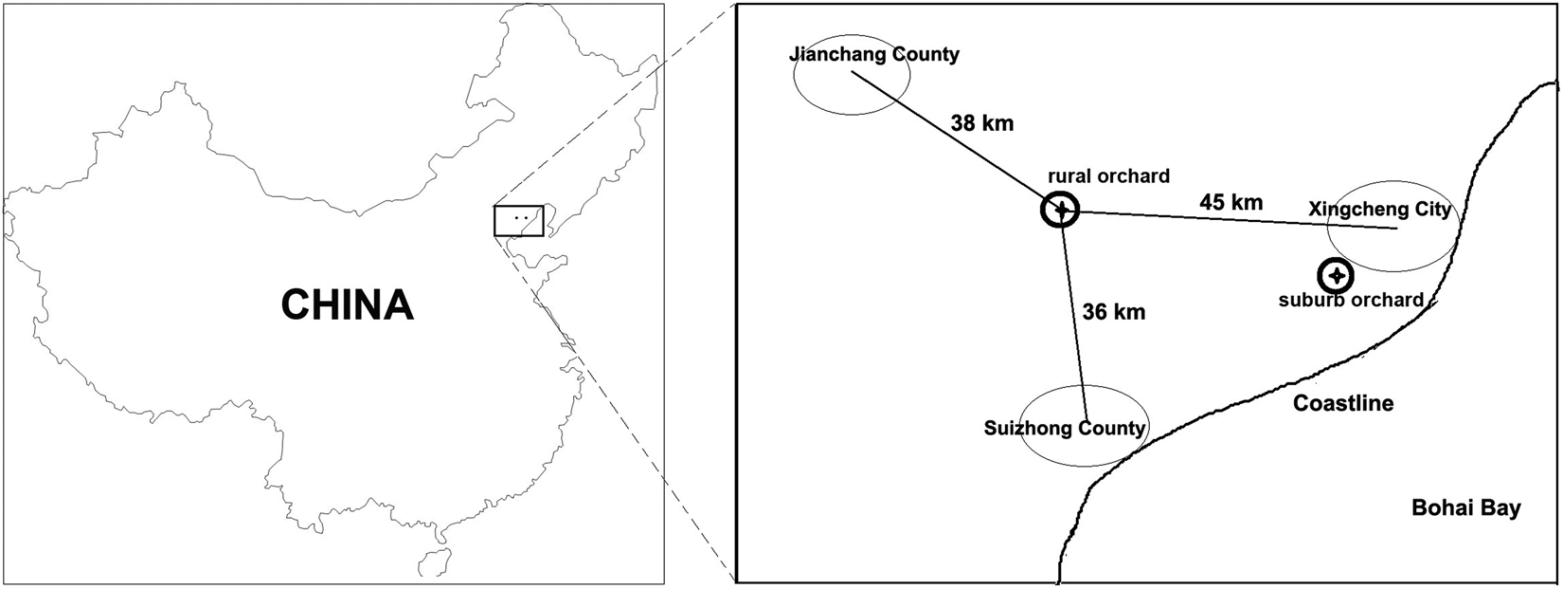
Locations of two orchards (rural orchard and peri-urban/suburb orchard) in Xingcheng, Liaoning, China, sampled in this study. Shen Youming created this figure. The simplified Chinese map was generated by modifying the map described by Zhang *et al*.^42^ by using Adobe PhotoShop (version CS5).

The objective of the present study was to evaluate the fungal community and diversity on the surface of apples at the point of harvest using fungal ITS1 sequencing. In our study, ‘Fuji’ apple samples were grown in two different geographical locations (Fig. 1)^42^. One orchard is far from the city (in a rural area), and the other is near the city. The samples were treated with the microbial community collection and analysed immediately after harvest. We focused on the following questions: (i) What are the fungal community and diversity on apple surfaces at the point of harvest? (ii) How do the fungal communities on the apple surfaces vary according to different factors of the rural/peri-urban environment? This study highlights the overall fungi communities on apple surfaces and compares the fungal differences of apples grown under various environmental conditions to provide a basis for further evaluation.

## Results

### ITS-based fungal community sequencing statistics

A total of 538,433 ITS reads were obtained from eight samples, with an average of 67,305 reads per sample. After quality control, we obtained 532,273 high-quality sequences (98.9% of total reads), with an average of 66,543 per sample. The length of the high-quality reads was mostly distributed between 200 and 300 bp. After filtering the rare OTUs (≤0.001% of the total sequences, ≤4 hits in this study), a total of 530,764 sequences were clustered in 421 identified OTUs taxonomies. The fungal OTUs’ taxonomic compositions and abundances are summarized in Table S1. The clustered OTUs of peri-urban samples (ranging from 204 to 220 OTUs and averaged 213 OTUs) were lower than those of rural samples (ranging from 215 to 263 OTUs and averaged 234 OTUs) (one-tail t-test, p = 0.05). The shared OTUs in samples were determined via a Venn diagram (Figure S1). A total of 294 OTUs overlapped between rural and peri-urban samples, occupying 69.8% of all OTUs (Figure S1a). A total of 55 OTUs were shared by all the samples (Figure S1b).

### Fungal diversity on the surface of apple fruits

Rarefaction curves, rank abundance curves, and alpha diversity indexes were applied in fungal alpha diversity analyses. The rarefaction curves and rank abundance curves all had a steep slope at the beginning, followed by long, flat tails, indicating that an increase in the amount of sequencing data would not yield more new OTUs (Figure S2). The rarefaction curves tended towards saturation, illustrating that the current sequencing depth was sufficient for fungal diversity investigation (Figure S2a). As shown in Figure S2b and Table S1, the majority of OTUs were detected with low abundance. The ACE and Simpson indexes of samples were calculated, and the results are summarized in Figure S3. The indexes of ACE were higher in rural samples than peri-urban samples, with a significantly difference in one-tail t-test (p = 0.042), indicating rural samples had an increase in fungal diversity.

### Overall characteristics of fungal community composition

**Figure 2:**
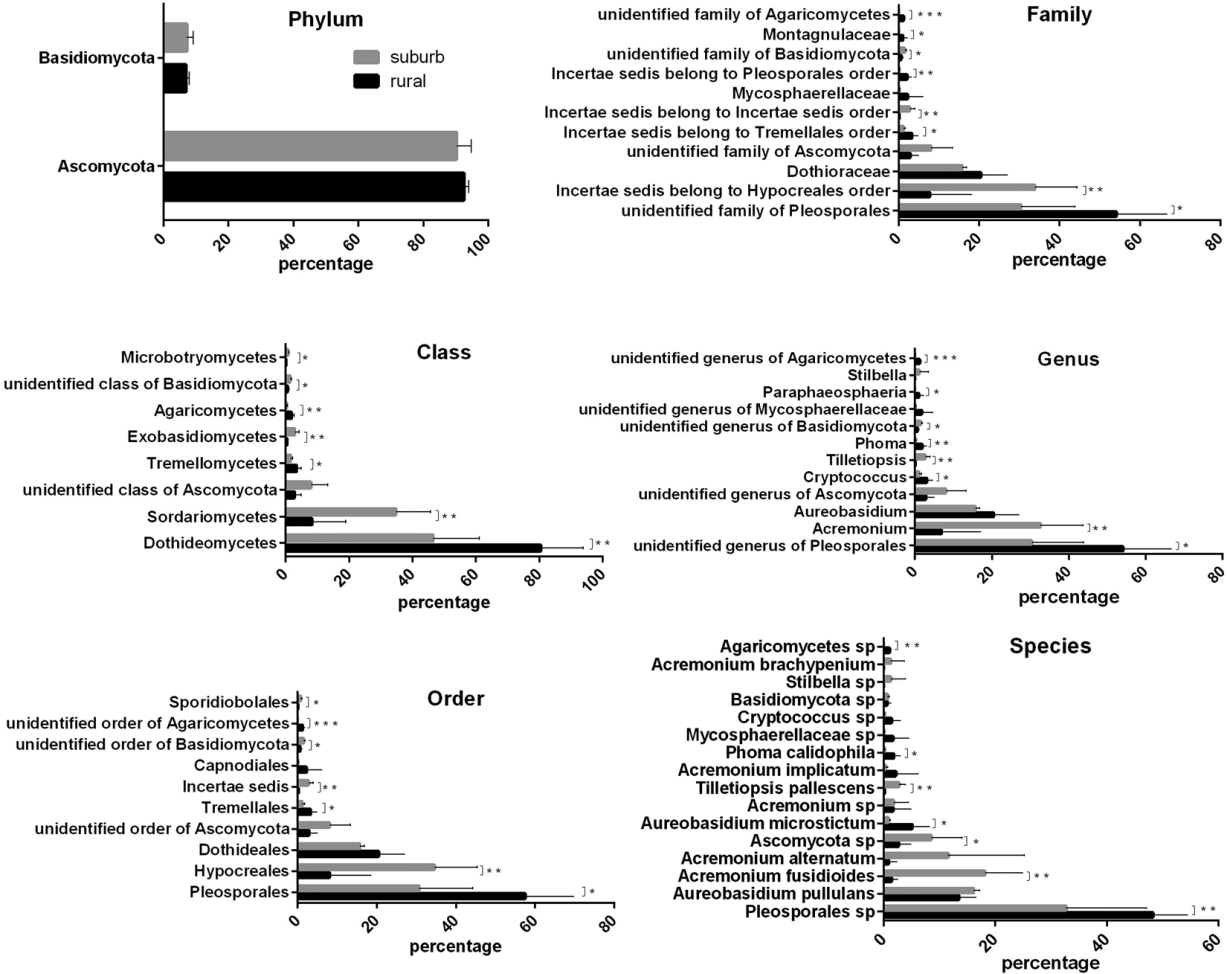
Distributions of predominant fungi (relative abundance ≥0.5%) at different taxonomic levels (phylum, class, order, family, and genus) of samples from two orchards. The significances were tested by one-tail Student’s t-test and marked as follow: p < 0.05 *p < 0.01 **p < 0.001 ***p ≥ 0.05 without a mark.

**Figure 3:**
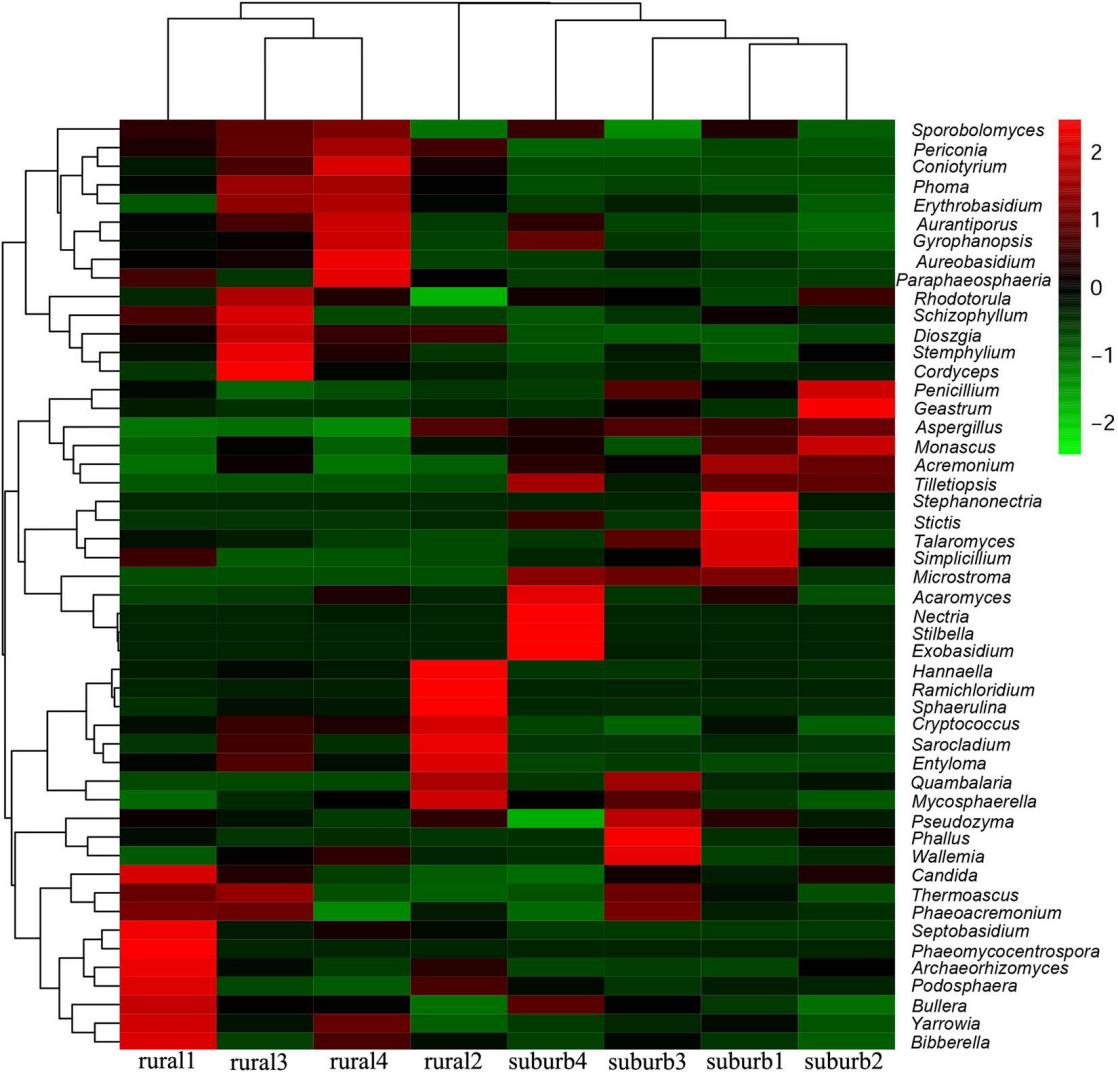
Heat map showing relative abundance of the 50 most dominant fungal genera in eight samples. Blue represents a fungus of relatively low abundance, and red represents a fungus of relatively high abundance. Cluster analyses of samples (vertical) and classification units (horizontal) were performed according to similarity.

A total of 360 fungal species belonging to four phyla, 17 classes, 50 orders, 79 families, and 111 genera were detected on the apple-surface samples. *Ascomycota* was the most abundant phylum, accounting for 90.2% of the total sequences. *Basidiomycota* was detected with relative lower abundance, accounting for 8.4% of the total sequences. We also detected the minor phyla *Chytridiomycota* and *Rozellomycota*. The distributions and percentages of predominant fungi at different classification levels (relative abundance ≥ 0.5%, including two phyla, 8 classes, 10 orders, 11 families, 12 genera, and 16 species) of rural and peri-urban samples are shown in Fig. 2. The relationship between fungal evolution and the fungal abundance of the two group samples were fully displayed by MEGA 5, and the classification taxa are shown in Figure S4. Additionally, a hierarchical tree of the overall fungal taxa composition at each taxonomic level was constructed by GraPhlAn, and the results are shown in Figure S5. The 20 most abundant taxa were marked by letters (A-T) and patterned using colour nodes (Figure S5). Moreover, the 50 most dominant fungal genera, coupled with cluster analysis are shown in the heat map (Fig. 3). Accordingly, certain genera were simultaneously enriched in samples; for example, *Nectria*, *Stilbella* and *Exobasidium* were enriched in sample suburb 4, and *Hannaella*, *Ramichloridium* and *Sphaerulina* were enriched in sample rural 2. Certain genera such as *Acremonium* and *Tilletiopsis* were enriched in samples from the peri-urban orchard, whereas the genera *Periconia*, *Coniothyrium* and *Phoma* were enriched in rural samples. The heat map also shows that samples from the same orchards tended to cluster together. Table S1 shows that the 5 most predominant genera on the apple surface included *Acremonium* (21.4%), *Aureobasidium* (17.8%), *Cryptococcus* (2.1%), *Tilletiopsis* (1.5%) and *Phoma* (1.0%), together accounting for 43.8% of the total sequences. As shown in Table S1, the majority of genera were detected with relatively low abundance.

### Association networks among fungal genera

**Figure 4:**
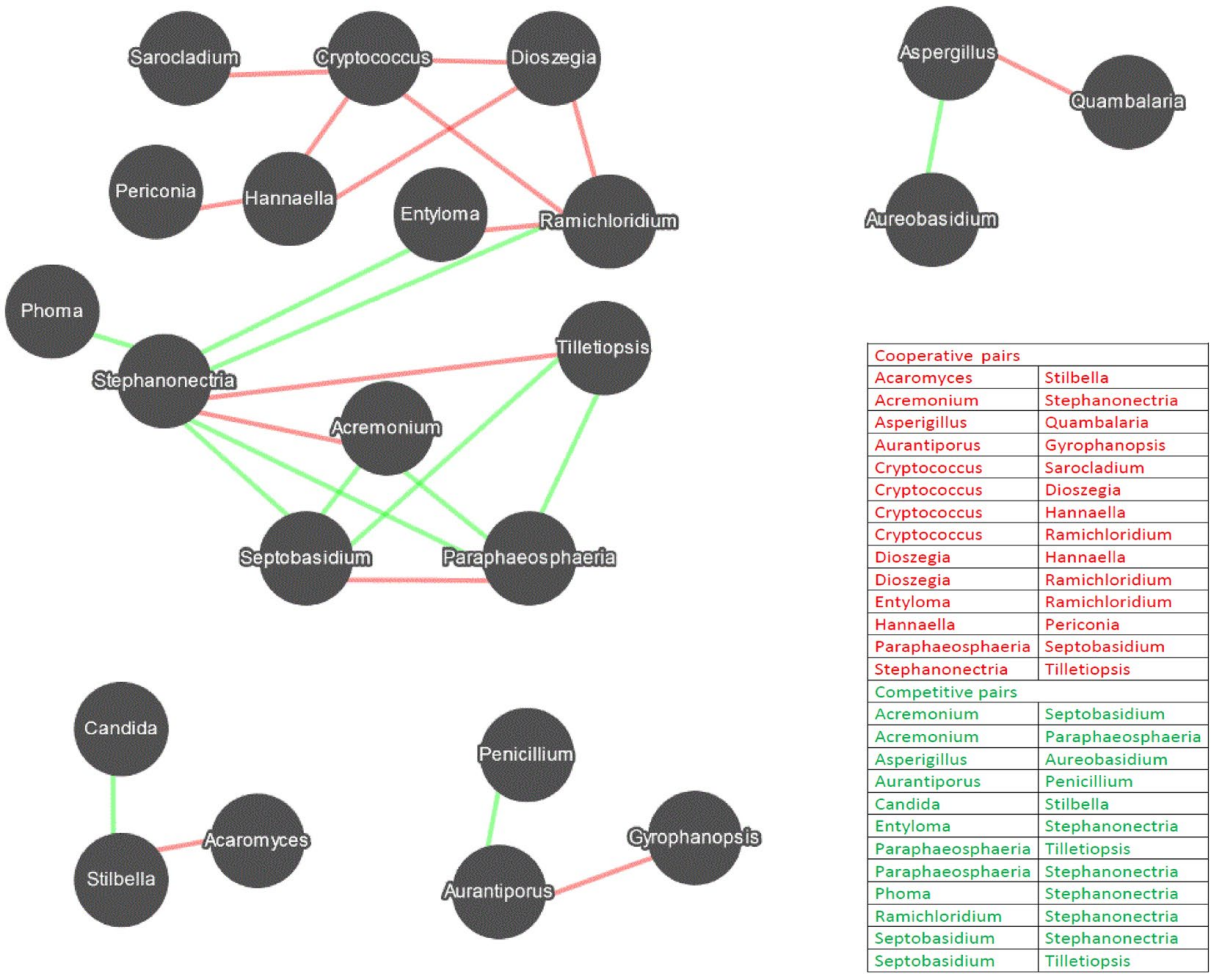
Network diagram of the 50 most dominant genera showing cooperative and competitive associations. Red represents cooperative association, and green represents competitive association.

Fungal co-occurrence networks among the 50 most abundant fungal genera are shown in Fig. 4. A total of 26 interaction-pairs related to 22 genera were obtained (rho > 0.8 and p < 0.01), mainly including *Acremonium, Aspergillus, Aureobasidium, Entyloma, Penicillium, Phoma, Stephanonetria*, and *Tilletiopsis*. There were 14 pairs of cooperative relationships and 12 pairs of competitive relationships. Among them, *Cryptococcus, Paraphaeosphaeria*, *Ramichloridium, Septobasidium* and *Stephanonectria* exhibited a higher degree of linkage (≥4 linkages) with other genera. As shown in Fig. 4, the genera *Cryptococcus, Dioszegia, Hannaella*, and *Ramichloridium* were generally observed to cooperate with other genera, whereas the genera *Acremonium*, *Paraphaeosphaeria*, *Septobasidium*, and *Stephanonectria* were observed to compete with other genera.

### Environment-related changes of fungal communities

**Figure 5:**
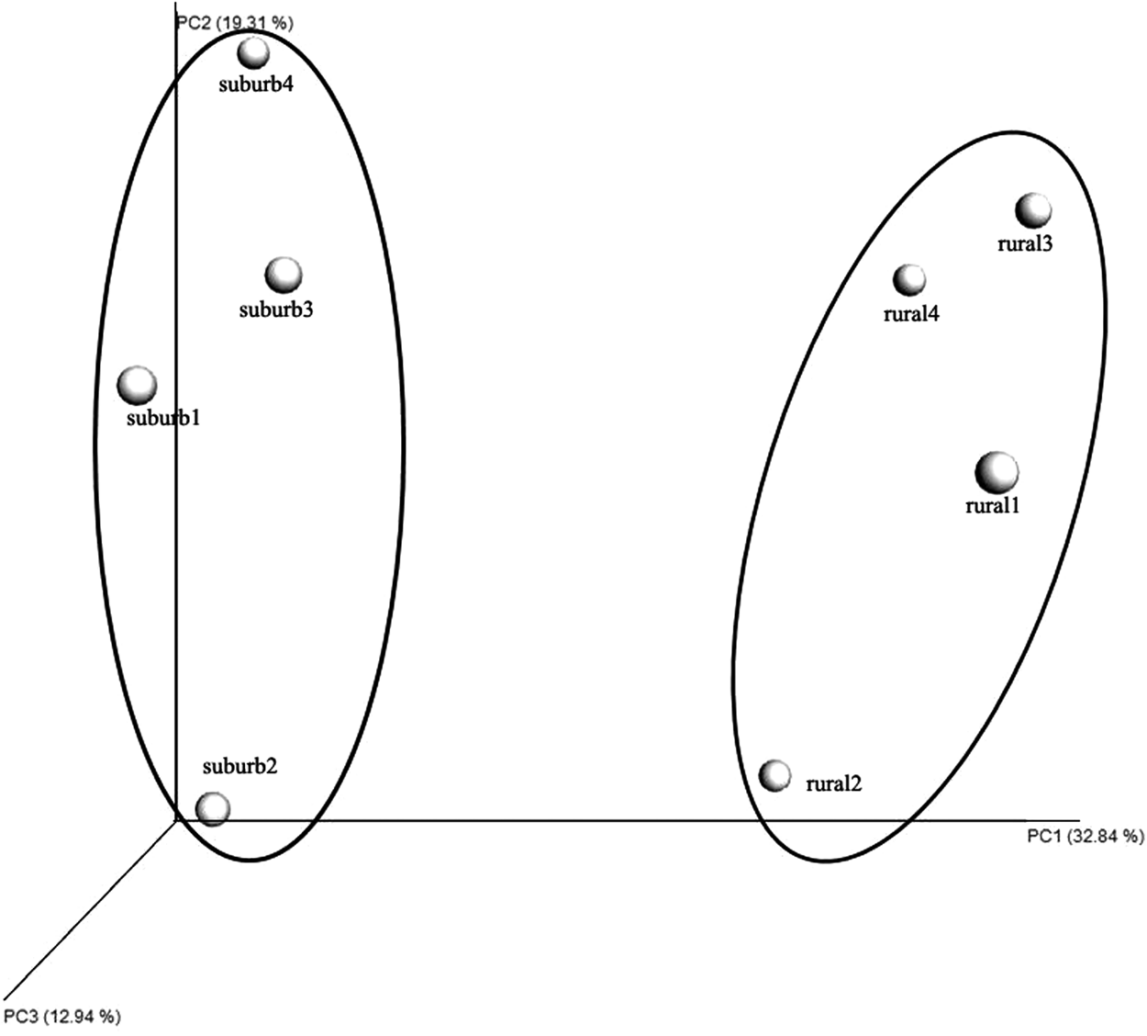
Beta diversity analysis was performed by principal coordinate analysis (PCoA) of the unweighted UniFrac distance among samples.

**Figure 6:**
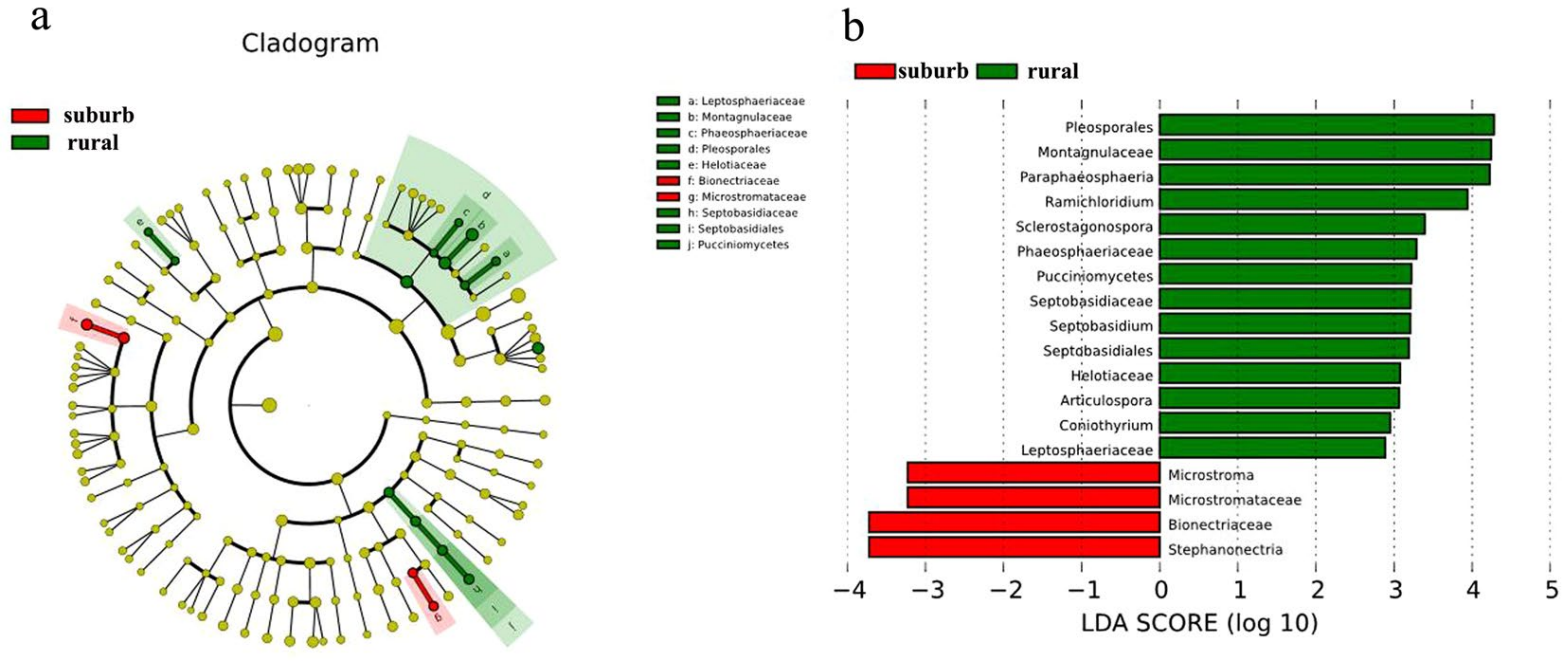
Comparison of fungal variations between rural and peri-urban samples using LEfSe. Differences in taxa are represented in red (peri-urban) and green (rural) colour. (**a**) Taxonomic cladogram presenting significant differences between groups. (**b**) Histogram of LDA scores (logarithmic LDA score > 2.0 and p < 0.05) for differentially abundant features between groups.

The beta diversity of fungal communities among samples was determined by unweighted UniFrac distances principal coordinates analysis (UniFrac PCoA) (Fig. 5). The UniFrac PCoA showed that samples from the two orchards had significant differences in fungal communities. These significant intergroup differences were further tested using several types of statistical analyses, including Adonis permutational multivariate analysis (Adonis/PERMANOVA), analysis of similarities (ANOSIM), and random forest analysis. The Adonis/PERMANOVA was scored with p = 0.013 (p < 0.05, in significant level), and the ANOSIM’s R statistical analysis was scored with R = 0.925, p = 0.01 (p < 0.05, in significant level). The random forest analysis indicated no estimated error or class error, further demonstrating the statistical significance of the fungal differences between samples. Cluster analysis of the 50 most dominant fungal genera indicated that samples from the same orchard trended to cluster together (Fig. 3). The significantly differently abundant taxa between samples from rural and peri-urban orchards were obtained using linear discriminant (LDA) effect size analysis (LEfSe), and the results are shown in Fig. 6. A cladogram presented 18 significantly different taxa between two groups. Fourteen taxonomies were remarkably enriched in rural samples including the two orders *Pleosporales* and *Septobasidiales*; the 5 families *Helotiaceae*, *Montagnulaceae*, *Leptosphaeriaceae*, *Phaeosphaeriaceae*, *Septobasidiaceae*; the class *Pucciniomycetes*; and the 6 genera *Septobasidium*, *Articulospora*, *Sclerostagonospora*, *Coniothyrium*, *Ramichloridium*, and *Paraphaeosphaeria*. The other four taxonomies were enriched in peri-urban samples including the 2 families *Bionectriaceae* and *Microstromataceae*, and the 2 genera *Stephanonectria* and *Microstroma*.

### Compositional variations of fungal genera between rural and peri-urban samples

The differential fungal compositions at the genus level were analysed using Student’s t-test, and the results are listed in Table 1. A total of 15 genera statistically differed in abundance between rural and peri-urban samples. Ten genera were enriched in rural samples, namely *Articulospora, Coniothyrium*, *Cryptococcus*, *Dioszegia*, *Entyloma*, *Paraphaeosphaeria*, *Periconia*, *Phoma*, *Sclerostagonospora* and *Septobasidium*. The five genera *Acremonium*, *Aspergillus*, *Microstroma*, *Penicillium*, and *Tilletiopsis* showed a significantly large distribution in peri-urban samples.

**Table 1:** Differential fungal compositions between groups at the genus level. Rural RA/Peri-urban RA represents the average relative abundance of rural/peri-urban samples at the genus level. The p-values were generated by one-tail Student’s t-test (p < 0.05 indicates significance).

Genus	Rural RA	SD	Peri-urban RA	SD	p-value
Acremonium	8.0898%	0.1250	32.6975%	0.1154	0.0083
Articulospora	0.0061%	0.0000	0.0000%	0.0000	0.0074
Aspergillus	0.1298%	0.0014	0.2980%	0.0003	0.0104
Coniothyrium	0.0172%	0.0001	0.0000%	0.0000	0.0240
Cryptococcus	3.1979%	0.0168	1.0963%	0.0059	0.0805
Dioszegia	0.0745%	0.0005	0.0056%	0.00001	0.0129
Entyloma	0.0614%	0.0005	0.0035%	0.0000	0.0286
Microstroma	0.0000%	0.0000	0.0304%	0.0002	0.0070
Paraphaeosphaeria	0.9895%	0.0095	0.0119%	0.0001	0.0428
Penicillium	0.0487%	0.0002	0.1371%	0.0008	0.0285
Periconia	0.1241%	0.0004	0.0094%	0.0001	0.0008
Phoma	1.9876%	0.0123	0.1874%	0.0007	0.0144
Sclerostagonospora	0.0057%	0.0000	0.0000%	0.0000	0.0008
Septobasidium	0.0586%	0.0006	0.0000%	0.0000	0.0442
Tilletiopsis	0.1737%	0.0011	2.7131%	0.0125	0.0032

## Discussion

The diversity of fungi on fresh apple surfaces is closely associated with fruit development, preservation and quality control^3–5^. A comprehensive and thorough investigation of fungal communities on apple surfaces is essential to improving fruit cultivation and disease prevention practices. Therefore, we evaluated the fungal community and their diversity on apple surfaces and identified the potential effects of peri-urban environments on fruit fungal signatures, which helps determine the complexity of fruit fungal communities, identify potential risks and improve fruit quality.

We investigated the overall fungal community using the Illumina sequencing technique. Compared with previous culture-based techniques^11–13^, the ITS1 sequencing technology is efficient and less labour-intensive and provides deeper insight into fungal community and diversity. The obtained OTU rarefaction curves (Figure S2a) tended towards saturation, indicating that the current sequencing depths were sufficient to detect the majority of fungi^43^. BLAST searching with 97% identification classified the fungi at the species level, in agreement with many previous studies^44,45^. We obtained a vast number of low-abundance OTUs (Table S1), which contributed the majority of fungal diversity. A total of 111 fungal genera belonging to 4 phyla were identified, indicating the remarkable fungal diversity on the apple surfaces. The present results pertaining to fungal profiles on apple surfaces are similar to those reported by Abdelfattah *et al*. regarding apple peel, who indicated comparable high-quality sequences ranging from 65,419 to 129,369 and identified similar fungi in 4 phyla and 24 predominant genera^31^. Previous reports on fruit fungal studies have mainly been conducted using culture-based techniques and have identified several fungi, such as *Alternaria*, *Aspergillus*, *Cladosporium* and *Penicillium*^11–13^. The most serious drawback of culture-based techniques is that they only quantify proportions of fungi belonging to specific cultivable taxonomic groups, which are mainly determined by the culture media used^28^. Significant differences in fungal profile have been reported in several studies of fruits^29,46^ and grains^47,48^, using similar DNA sequencing methods. Abdelfattah *et al*. reported on fungal communities on strawberry, obtaining 218,164 high-quality sequences, and identifying fungi mainly belonging to the genera *Botrytis* and *Cladosporium*^29^. Xing *et al*. reported on fungal communities on peanut kernels, obtaining an average of 36,718 reads (belonging 196 OTUs) and identifying fungi belonging to 37 genera^48^. As discussed in the literature, such fungal differences might be related to plant species^28^, environmental factors^27,48^ and farming systems^49^. Moreover, differences in plant pattern^50^, sample size^51^, sampling time point^27^, and extraction method can potentially affect fungal diversity.

*Ascomycota* was the predominantly detected phylum, which was present in all samples, coinciding with the results of Abdelfattah *et al*.^31^. *Ascomycota* is the largest phylum of fungi kingdom, which contains approximately 64,000 fungal species^52^. *Basidiomycota* was detected with relative lower abundance. Genera of *Tilletiopsis*, *Cryptococcus* and *Gyrophanopsis* were mainly detected in *Basidiomycota*. *Tilletiopsis* (mainly enriched in peri-urban samples) might cause white haze disease in apple^53^. *Cryptococcus* (mainly enriched in rural samples) in particular contains several potential pathogenic species (such as *Cryptococcus gattii* and *Cryptococcus neoformans*), which are agents of human Cryptococcosis^54^. The 50 most predominant genera shown in the heat map may play important roles in maintaining fruit fungal stability. As shown in Fig. 3, the presence of potential pathogenic fungi may be related to a variety of plant diseases or fruit post-harvest rots; these fungi include *Acremonium*^55^, *Aspergillus*^10^, *Entyloma*^56^, *Exobasidium*^57^, *Microstroma*^58^, *Mycosphaerella*^59^, *Nectria*^55^, *Penicillium*^10^, *Phoma*^60^, *Podosphaera*^60^, *Ramichloridium*^61^, *Sarocladium*^62^, *Stemphylium*^63^ and *Tilletiopsis*^53^. In particular, *Acremonium* (mainly enriched in peri-urban samples) might be associated with black spot disease in apple fruit^55,64^; *Penicillium* and *Aspergillus* (mainly enriched in peri-urban samples) might be associated with apple post-harvest deterioration and rot^10^; *Mycosphaerella* causes apple plant diseases^59^; *Nectria* might cause apple tree canker^65^; *Phoma* (mainly enriched in rural samples) causes apple rot^66^; and *Podosphaera* causes apple mildew^60^. The potential endophytes living in plant tissues might play essential roles in disease control, particularly in *Aureobasidium*^67^, *Paraphaeosphaeria*^68^, *Stephanonectria*^69^, and *Talaromyces*^70,71^. *Aureobasidium* (enriched mainly in rural samples), covered with a slimy mass of spores, has been used in the biological control of plant and post-harvest diseases^67,72^. Potential entomopathogenic fungi genera, including *Cordyceps*^73^ and *Simplicillium*^74^, are parasitic toward insects and other arthropods and are regarded as beneficial for pest control. Potential human foodborne pathogenic fungi include *Aspergillus*^75^, *Candida*^76^, *Cryptococcus*^54^, *Penicillium*^75^, *Phaeoacremonium*^77^, *Rhodotorula*^78^, and *Wallemia*^79^. Compared with apple fungi profiles reported in previous studies^31,46^, we observed more detailed fungal communities and classifications. However, the relationships between the obtained fungal communities and those described by Abdelfattah *et al*.^31^ were difficult to define because many factors influence fungi formation or sample isolation. To understand the full community, its diversity and the corresponding ecological interactions, fungi on apple surfaces require further study.

Inter-genus interactions are extremely important in shaping fungal dynamics. The network analysis results shown in Fig. 4 clearly demonstrate potential intra-genus relationships. The entangled interactions were difficult to identify because one genus can directly or indirectly influence another genus. Despite the complicated networks of many fungal genera, we compared our results with those of several other related reports. We observed that *Stephanonectria* was competitive with both *Entyloma* and *Phoma*. *Stephanonectria* was reported as an endophytic fungi^69,80^, whereas *Entyloma*^56^ and *Phoma*^60^ were reported as plant pathogens. From this point of view, it is reasonable to propose that *Stephanonectria* is a beneficial symbiont and might potentially protect apple fruit from diseases caused by the pathogens *Entyloma* and *Phoma*. The genus *Paraphaeosphaeria* was observed to be competitive with *Acremonium*. *Paraphaeosphaeria* was reported to be an endophytic fungi and yielded antifungal metabolites^68,81^. *Acremonium* was reported to cause black spot disease in apple fruit^64^. This result indicates that *Paraphaeosphaeria* might be used as a bio-control agent for controlling *Acremonium*-caused black spot disease. The genus *Aureobasidium* was used in the biological control of plant and post-harvest diseases^67,72^. We observed that *Aureobasidium* was competitive with *Aspergillus*, consistent with previous reports indicating competition between *Aureobasidium* and *Aspergillus*^82,83^. These results generally indicate that protective fungi guard fruit against pathogenic fungi. In fact, the inter-genus interactions obtained in our study require further investigation and verification. The present network analysis provides a basis for further fungal interactions analysis and fruit disease bio-control.

Environmental-related changes of fungal communities were tested by comparative analyses. Using Student’s t-test, we found that OTU numbers and ACE indexes of rural samples were higher than those of peri-urban samples. These results indicate that rural samples share a more diverse fungal community than peri-urban samples do. Similarly, several studies reported that organic-labelled fruits and vegetables have a greater richness of microbial OTUs than conventionally labelled produce^28,32^. From a bacterial point of view, a more diverse community might represent a more stable, healthier ecosystem^28,84^. The obtained results indicate that rural areas might provide a healthier environment for apple growing. Therefore, the rural orchard environments examined in this study are beneficial for apple fungal diversity.

Beta diversity revealed significantly different fungal communities between rural and peri-urban samples. A total of 294 OTUs (69.8% of 421 OTUs) overlapped between samples from the two different orchards (Figure S1a), indicating the existence of considerable amounts of distinguished OTUs (approximately 30%). The results of the beta diversity analysis (Fig. 5) and the heat map of the 50 most dominant fungal genera (Fig. 3) tend to cluster samples from identical orchards. Additionally, these results are generally consistent with the results of the Adonis/PERMANOVA, ANOSIM and random forests analyses. LEfSe results showed significantly different classification taxa between rural and peri-urban samples (Fig. 6). Overall, these results indicate a significant difference in fungal communities on the apple surfaces from two types of peri-urban and rural environments. Previous studies have indicated that fungal structural differences might be related to factors such as fruit species^28^, farming practices^19^, and climatic or environmental conditions^32^. In the present study, different levels of environment pollution in peri-urban and countryside areas were regarded as one of the most distinguishing conditions, over farming practices, climatic conditions and others. We observed that the fungal differences identified on apple surfaces from peri-urban and rural orchards might be due to different levels of pollution in their respective environments. Further analysis of fungal communities on produce and their diversity in several environments should be tested to confirm this theoretical mechanism.

Different fungal compositions at the rural and peri-urban genus level (Table 1) indicate that environmental factors significantly affect fungal compositions. A total of 10 genera were enriched in rural samples, whereas a total of 5 genera were enriched in peri-urban samples. Notably, the genera enriched in rural samples were reported as protective fungi, mainly *Coniothyrium*^85^, *Paraphaeosphaeria*^68,81^, *Periconia*^86^ and *Sclerostagonospora*^87^. The genus *Coniothyrium* might have the ability to control fruit disease. For example, the *Coniothyrium minitans* species has been widely reported as a bio-control agent against the plant-pathogenic fungus *Sclerotinia sclerotiorum*^88^. Genera of *Paraphaeosphaeria*^68^, *Periconia*^86^ and *Sclerostagonospora*^87^ have been reported to be endophytic fungi. *Entyloma*^56^ and *Phoma*^60^ (enriched rural samples) have been reported to show pathogenic features*. Articulospora, Dioszegia* and *Septobasidium* have rarely been studied. In contrast, the fungal genera enriched in peri-urban samples have all been reported to exhibit pathogenic features and are associated with fruit corruption. Specifically, *Acremonium* might be associated with black spot disease in apple fruit^55,64^. *Aspergillus* and *Penicillium* are the major microbes causing fruit deterioration and the accumulation of mycotoxins (mainly patulin, ochratoxins and aflatoxins)^10^. *Tilletiopsis* might cause white haze disease in apple^53^. *Microstroma* has been reported to cause walnut downy leaf spot^58^. As a result, we observed significantly different fungi genera enriched in peri-urban and rural samples. Certain fungal genera enriched in rural samples have been reported to exhibit protective functions. Conversely, the genera enriched in peri-urban samples have mainly been reported to exhibit pathogenic features and are associated with fruit corruption. Therefore, the rural environment provides certain advantages in maintaining healthier fungal communities on apple surfaces. Apple are grown in peri-urban environments carry potential microbiological risks.

In conclusion, fungal ITS1 region sequencing techniques were successfully applied to the study of fungal community and diversity on apple surfaces. We observed high fungal diversity on the surfaces of apples, which enhances our understanding of fungal communities on apples. Furthermore, several potentially protective and pathogenic genera were identified. Co-occurrence relationships indicate that protective fungi guard plants against pathogenic fungi. The diversity analyses reveal the presence of significant differences in fungal community and diversity between samples from rural and peri-urban orchards. The rural samples maintained more protective fungal genera on apple surfaces, whereas the peri-urban planted apples carried potential microbiological risks. We observed that the fungal differences identified on apple surfaces from peri-urban and rural orchards were due to different levels of pollution in their respective environments. However, further studies should be valuable in confirming this theoretical mechanism. This study will help to reveal the complexity of fungal communities on apple fruit and shed light on ways to improve fruit disease prevention and quality control.

## Methods

### Orchard characteristics and sample treatment

This study was conducted in two ‘Fuji’ apple orchards: the first, located near the city comprises approximately 5 acres of flat land at the Hot Springs Experimental Base of the Institute of Pomology, Chinese Academy of Agricultural Sciences (E: 120.72, N: 40.61); the second, located in a rural area, spans approximately 10 acres on the sunny side of a hilly, gentle slope, approximately 40 km away from the city (E: 120.14, N: 40.65) (Fig. 1)^42^. A total of 24 trees selected in our study were evenly distributed between the two orchards (not on the border). The orchards were supervised under conventional management; the pesticides λ-cyhalothrin, pyridaben and carbendazim were utilized within the recommended concentrations for insect and disease control. Four replicates of apple samples were collected from each orchard on October 10^th^, 2016.

### Sample preparation

Eight samples (4 replicates × 2 orchards) of apple-surface microbial DNA were collected after harvest. The microbial samples were gathered by wiping or swabbing each apple with a pre-moistened cotton swab. Swab samples were collected and stored at −40 °C for less than two weeks before microbial DNA extraction. Microbial genomic DNA from swab was extracted using the MoBio Power Water® DNA Isolation kit (MoBio Laboratories, Inc., Carlsbad, CA, USA) according to the manufacturer’s instructions. The concentration and molecular size of DNA extraction were measured using an NC 2000 spectrophotometer (Thermo Scientific Fisher, Waltham, MA, USA) and 1.0% agarose gel electrophoresis, respectively. The spectrophotometric A260:A280 ratios were above 1.8, and DNA concentrations were above 80 ng/µL, indicating that the DNA extractions were sufficient for subsequent analysis. Finally, the DNA extractions were stored at −80 °C until further use.

### DNA amplification and sequencing

The fungal ITS1 was PCR-amplified according to processes described previously^89^. The primer pairs were ITS5F: 5′-GGAAGTAAAAGTCGTAACAAGG-3′ and ITS1R: 5′-GCTGCGTTCTTCATCGATGC-3′. The PCR incubation system contained 5 U of DNA polymerase (Pyrobest TaKaRa, Japan), 15 pmol of both primers, 2.5 mM of dNTP mixture, 10 μL of 10x Buffer II, and 40 ng of template DNA for a total volume of 25 μL. Next, a PCR series was performed using an ABI 9600 instrument under the following conditions: first denaturation at 94 °C for 4 min; 25 cycles of denaturation at 98 °C for 45 s, annealing at 60 °C for 45 s, extension at 72 °C for 45 s; and final elongation at 72 °C for 8 min. The amplicons were purified with Agencourt AMPure Beads (Beckman Coulter, Indianapolis, IN) by following the recommended procedures. The obtained ITS amplicons were sent to Shanghai Personal Biotechnology Co., Ltd. (Shanghai, China), and sequenced on an Illumina MiSeq paired-end sequencing platform.

### Data quality control and analysis

Data quality control and analysis were mainly conducted using the software package QIIME (http://qiime.org/)^90^. The raw data were first filtered by removing sequences of base quality ≤ Q20, without primers, and with terminals mismatched with both primers (by FLASH v1.2.7, http://ccb.jhu.edu/software/FLASH/)^91^. To obtain high-quality sequences, we discarded low-quality sequences, including sequences shorter than 150 bp, sequences with any ambiguous bases, sequences containing more than 6 mononucleotide repeats, sequences with a Phred score below 25, and chimeric sequences (by QIIME). These high-quality sequences were clustered into operational taxonomic units (OTUs) by the QIIME-uclust OTU-picking workflow at 97% sequence identity^44,92^. OUT taxa were assigned by BLAST searching against the UNITE database using the best hits (Release 5.0, https://unite.ut.ee/)^92^. The OTU taxonomy was simplified by removing rare OTUs (≤0.001% of the total sequences) to reduce the complexity of the subsequent analysis^93^. Qualitative and quantitative information about the OTUs is listed in Table S1 and was used for subsequent analysis.

Based on the OTU results, rarefaction curves and rank abundance curves^43^ were plotted to explain both the richness and evenness of the fungi community. The alpha diversity indexes, including ACE^94^ and Simpson^95^ indexes, were calculated to investigate the sequencing depth and fungal diversity. Student’s t-test and ANOVA were used to identify significant differences in alpha indexes and differential fungal compositions at the genus level of different groups (p < 0.05). Venn diagrams were generated by the mothur software package to identify shared and unique OTUs among groups^92^. The relationship between fungal evolution and taxonomy abundance was displayed using MEGA 5 (http://ab.inf.uni-tuebingen.de/software/megan5/); a hierarchical tree was constructed using GraPhlAn^96^. Beta diversity analysis was conducted to investigate the similarity of fungal communities among samples using UniFrac PCoA^97^. The differences in multi-group UniFrac distances for pairwise comparisons between groups were determined using Student’s t-test and the Monte Carlo permutation test with 1,000 permutations, and visualized using boxplots (by QIIME)^98^. Adonis permutational multivariate analysis (Adonis/PERMANOVA)^99^, analysis of similarities (ANOSIM)^100^, and random forest analysis^101^ were performed to evaluate the fungal similarity among samples. LEfSe was used to detect differentially abundant taxa using the online Galaxy workflow framework (logarithmic LDA score > 2.0 and p < 0.05) (http://huttenhower.sph.harvard.edu/galaxy/)^102^. The Spearman rank correlation coefficients of the 50 most abundant genera were calculated to observe the potential cooperative and competitive network among the genera using the mothur software package and visualized by Cytoscape (rho > 0.8 and p < 0.01) (http://www.cytoscape.org/)^103^.

### Data availability

All data generated or analysed in this study are included in this published article (and its Supplementary Information files).

## Electronic supplementary material

Supplementary File
