# Author Correction: Differentiated surface fungal communities at point of harvest on apple fruits from rural and peri-urban orchards

**DOI:** 10.1038/s41598-018-23387-2

**Published:** 2018-03-19

**Authors:** Youming Shen, Jiyun Nie, Zhixia Li, Haifei Li, Yonglong Wu, Yafeng Dong, Jianyi Zhang

**Affiliations:** 1 Institute of Pomology, Chinese Academy of Agricultural Sciences, Xingcheng, 125100 P.R. China; 2 Laboratory of Quality & Safety Risk Assessment for Fruit (Xingcheng), Ministry of Agriculture, Xingcheng, 125100 P.R. China; 3 Quality Inspection and Test Center for Fruit and Nursery Stocks (Xingcheng), Ministry of Agriculture, Xingcheng, 125100 P.R. China

Correction to: *Scientific Reports* 10.1038/s41598-017-17436-5, published online 01 February 2018

The Acknowledgements section in this Article is incomplete.

“This work was supported by the National Program for Quality and Safety Risk Assessment of Agricultural Products of China (No. GJFP2016003) and the Agricultural Science and Technology Innovation Program of Chinese Academy of Agricultural Sciences (No. CAAS-ASTIP). We acknowledge Shanghai Personal Biotechnology Co., Ltd. (Shanghai, China) for their help in sequencing and bioinformatics analysis.”

should read:

“This work was supported by the National Program for Quality and Safety Risk Assessment of Agricultural Products of China (No. GJFP2016003), the Agricultural Science and Technology Innovation Program of Chinese Academy of Agricultural Sciences (No. CAAS-ASTIP), and the Earmarked Fund for China Agriculture Research System (No. CARS-27). We acknowledge Shanghai Personal Biotechnology Co., Ltd. (Shanghai, China) for their help in sequencing and bioinformatics analysis.”

