# Supplementary material for: Differentiated surface fungal communities at point of harvest on apple fruits from rural and peri-urban orchards
Source: Sci Rep. 2018 Feb 1;8:2165. doi: 10.1038/s41598-017-17436-5 (PMC5794916; doi:10.1038/s41598-017-17436-5)

Differentiated surface fungal communities at point of harvest on apple fruits from rural and peri-urban orchards

Shen Youming, Nie Jiyun*, Li Zhixia, Li Haifei, Wu Yonglong, Dong Yafeng, Zhang Jianyi

*Corresponding author

Table S1. Fungal OTUs hits and taxonomic compositions.

OTU ID	Rural 1	Rural 2	Rural 3	Rural 4	Suburb 1	Suburb 2	Suburb 3	Suburb 4	taxonomy
OTU001	35869	35276	28636	28331	15997	16310	35978	18473	k__Fungi; p__Ascomycota; c__Dothideomycetes; o__Pleosporales; f__unidentified; g__unidentified; s__Pleosporales sp
OTU002	6623	8030	11199	9955	11095	9830	11403	10419	k__Fungi; p__Ascomycota; c__Dothideomycetes; o__Dothideales; f__Dothioraceae; g__Aureobasidium; s__Aureobasidium pullulans
OTU003	387	1463	1706	154	12610	8892	8582	18073	k__Fungi; p__Ascomycota; c__Sordariomycetes; o__Hypocreales; f__Incertae sedis; g__Acremonium; s__Acremonium fusidioides
OTU004	41	252	2002	8	18974	11057	304	315	k__Fungi; p__Ascomycota; c__Sordariomycetes; o__Hypocreales; f__Incertae sedis; g__Acremonium; s__Acremonium alternatum
OTU005	2813	1472	2942	6316	580	392	812	551	k__Fungi; p__Ascomycota; c__Dothideomycetes; o__Dothideales; f__Dothioraceae; g__Aureobasidium; s__Aureobasidium microstictum
OTU006	124	894	2656	309	500	6935	105	5804	k__Fungi; p__Ascomycota; c__unidentified; o__unidentified; f__unidentified; g__unidentified; s__Ascomycota sp
OTU007	16	1746	690	584	2787	3412	2297	888	k__Fungi; p__Ascomycota; c__unidentified; o__unidentified; f__unidentified; g__unidentified; s__Ascomycota sp
OTU008	39	179	122	60	2134	2010	709	2430	k__Fungi; p__Basidiomycota; c__Exobasidiomycetes; o__Incertae sedis; f__Incertae sedis; g__Tilletiopsis; s__Tilletiopsis pallescens
OTU009	4	55	4280	4	61	798	3771	124	k__Fungi; p__Ascomycota; c__Sordariomycetes; o__Hypocreales; f__Incertae sedis; g__Acremonium; s__Acremonium sp
OTU010	97	300	5409	121	59	207	597	14	k__Fungi; p__Ascomycota; c__Sordariomycetes; o__Hypocreales; f__Incertae sedis; g__Acremonium; s__Acremonium implicatum
OTU011	15	38	131	22	1036	376	64	4013	k__Plantae; p__unidentified; c__unidentified; o__unidentified; f__unidentified; g__unidentified; s__Plantae sp
OTU012	509	657	2077	1565	120	82	202	126	k__Fungi; p__Ascomycota; c__Dothideomycetes; o__Pleosporales; f__Incertae sedis; g__Phoma; s__Phoma calidophila
OTU013	0	2	1	2	465	506	727	682	k__Fungi; p__Basidiomycota; c__unidentified; o__unidentified; f__unidentified; g__unidentified; s__Basidiomycota sp
OTU014	82	3927	62	354	3	12	31	195	k__Fungi; p__Ascomycota; c__Dothideomycetes; o__Capnodiales; f__Mycosphaerellaceae; g__unidentified; s__Mycosphaerellaceae sp
OTU015	152	2424	953	315	52	16	85	134	k__Fungi; p__Basidiomycota; c__Tremellomycetes; o__Tremellales; f__Incertae sedis; g__Cryptococcus; s__Cryptococcus sp

OTU016	9	153	514	4	24	740	544	383	k__Fungi; p__Ascomycota; c__Sordariomycetes; o__Hypocreales; f__Incertae sedis; g__Acremonium; s__Acremonium sp
OTU017	185	256	1044	181	239	531	285	762	k__Fungi; p__Basidiomycota; c__unidentified; o__unidentified; f__unidentified; g__unidentified; s__Basidiomycota sp
OTU018	0	5	0	2	4	0	0	3426	k__Fungi; p__Ascomycota; c__Sordariomycetes; o__Hypocreales; f__Incertae sedis; g__Stilbella; s__Stilbella sp
OTU019	0	10	0	2	1	3288	3	0	k__Fungi; p__Ascomycota; c__Sordariomycetes; o__Hypocreales; f__Incertae sedis; g__Acremonium; s__Acremonium brachypenium
OTU020	847	502	642	885	0	0	6	14	k__Fungi; p__Basidiomycota; c__Agaricomycetes; o__unidentified; f__unidentified; g__unidentified; s__Agaricomycetes sp
OTU021	599	338	69	1407	4	6	5	2	k__Fungi; p__Ascomycota; c__Dothideomycetes; o__Pleosporales; f__Montagnulaceae; g__Paraphaeosphaeria; s__Paraphaeosphaeria sp
OTU022	31	52	59	126	307	856	362	357	k__Fungi; p__Basidiomycota; c__Microbotryomycetes; o__Sporidiobolales; f__Incertae sedis; g__unidentified; s__Incertae sedis sp
OTU023	495	285	752	605	0	0	4	15	k__Fungi; p__Basidiomycota; c__Tremellomycetes; o__Tremellales; f__Incertae sedis; g__Cryptococcus; s__Cryptococcus sp
OTU024	38	125	1525	5	9	178	146	3	k__Fungi; p__Ascomycota; c__Sordariomycetes; o__Hypocreales; f__Incertae sedis; g__Acremonium; s__Acremonium implicatum
OTU025	3	74	134	248	322	201	328	585	k__Fungi; p__Basidiomycota; c__Tremellomycetes; o__Filobasidiales; f__unidentified; g__unidentified; s__Filobasidiales sp
OTU026	8	1344	545	17	1	1	5	23	k__Fungi; p__Ascomycota; c__Sordariomycetes; o__Hypocreales; f__Incertae sedis; g__Sarocladium; s__Sarocladium strictum
OTU027	3	71	22	21	146	134	1005	267	k__Fungi; p__Ascomycota; c__Sordariomycetes; o__Hypocreales; f__Incertae sedis; g__Acremonium; s__Acremonium fusidioides
OTU028	73	182	210	263	147	167	104	233	k__Fungi; p__Basidiomycota; c__Tremellomycetes; o__Tremellales; f__Incertae sedis; g__Cryptococcus; s__Cryptococcus sp
OTU029	185	153	97	107	50	263	252	285	k__Fungi; p__Basidiomycota; c__Tremellomycetes; o__Tremellales; f__Incertae sedis; g__Cryptococcus; s__Cryptococcus aureus
OTU030	0	0	56	6	1322	3	3	0	k__Fungi; p__Ascomycota; c__Sordariomycetes; o__Hypocreales; f__Incertae sedis; g__Acremonium; s__Acremonium alternatum
OTU031	0	0	0	0	3	13	6	31	k__Fungi; p__Basidiomycota; c__unidentified; o__unidentified; f__unidentified; g__unidentified; s__Basidiomycota sp
OTU032	112	76	264	326	58	23	73	221	k__Fungi; p__Basidiomycota; c__Agaricomycetes; o__Polyporales; f__Polyporaceae; g__Aurantiporus; s__Aurantiporus alborubescens

OTU033	6	116	46	7	961	7	5	29	k__Fungi; p__Basidiomycota; c__Tremellomycetes; o__Tremellales; f__Incertae sedis; g__Cryptococcus; s__Cryptococcus victoriae
OTU034	1	1	763	7	0	0	355	1	No blast hit
OTU035	97	203	96	96	6	20	18	29	k__Fungi; p__Basidiomycota; c__Exobasidiomycetes; o__Exobasidiales; f__unidentified; g__unidentified; s__Exobasidiales sp
OTU036	41	16	177	89	143	149	81	21	k__Fungi; p__Ascomycota; c__Sordariomycetes; o__Hypocreales; f__Nectriaceae; g__unidentified; s__Nectriaceae sp
OTU037	7	0	0	3	813	0	20	6	k__Fungi; p__Ascomycota; c__Sordariomycetes; o__Hypocreales; f__Incertae sedis; g__Acremonium; s__Acremonium sp
OTU038	12	42	768	11	0	1	0	0	k__Fungi; p__Ascomycota; c__Sordariomycetes; o__Hypocreales; f__Incertae sedis; g__Acremonium; s__Acremonium implicatum
OTU039	9	90	23	8	180	188	116	155	k__Fungi; p__Ascomycota; c__Eurotiomycetes; o__Eurotiales; f__Trichocomaceae; g__Aspergillus; s__Aspergillus subversicolor
OTU040	5	8	26	2	123	51	8	538	k__Plantae; p__unidentified; c__unidentified; o__unidentified; f__unidentified; g__unidentified; s__Plantae sp
OTU041	2	710	3	9	0	0	0	2	k__Fungi; p__Ascomycota; c__Dothideomycetes; o__Capnodiales; f__Mycosphaerellaceae; g__Ramichloridium; s__Ramichloridium luteum
OTU042	0	0	0	0	650	26	1	2	k__Fungi; p__Ascomycota; c__Sordariomycetes; o__Hypocreales; f__Bionectriaceae; g__Stephanonectria; s__Stephanonectria keithii
OTU043	52	31	86	166	26	12	41	149	k__Fungi; p__Basidiomycota; c__Agaricomycetes; o__Polyporales; f__Meruliaceae; g__Gyrophanopsis; s__Gyrophanopsis polonensis
OTU044	0	0	0	0	0	0	0	581	k__Fungi; p__Ascomycota; c__Sordariomycetes; o__Hypocreales; f__Hypocreaceae; g__unidentified; s__Hypocreaceae sp
OTU045	14	76	46	14	1	2	5	2	k__Fungi; p__Basidiomycota; c__Exobasidiomycetes; o__Entylomatales; f__Entylomataceae; g__Entyloma; s__Entyloma gaillardianum
OTU046	35	108	161	39	63	56	27	17	k__Fungi; p__Ascomycota; c__Saccharomycetes; o__Saccharomycetales; f__unidentified; g__unidentified; s__Saccharomycetales sp
OTU047	48	64	63	41	73	57	96	34	k__Fungi; p__Basidiomycota; c__Ustilaginomycetes; o__Ustilaginales; f__Ustilaginaceae; g__Pseudozyma; s__Pseudozyma rugulosa
OTU048	5	294	52	26	15	16	17	21	k__Fungi; p__Ascomycota; c__Dothideomycetes; o__Capnodiales; f__Mycosphaerellaceae; g__Sphaerulina; s__Sphaerulina sp
OTU049	96	24	47	21	73	57	80	27	k__Fungi; p__Ascomycota; c__Sordariomycetes; o__Coniochaetales; f__Coniochaetaceae; g__unidentified; s__Coniochaetaceae sp

OTU050	158	78	108	82	1	4	0	0	k__Fungi; p__Ascomycota; c__Lecanoromycetes; o__Peltigerales; f__Pannariaceae; g__unidentified; s__Pannariaceae sp
OTU051	26	6	33	57	18	18	53	21	k__Fungi; p__Ascomycota; c__Sordariomycetes; o__unidentified; f__unidentified; g__unidentified; s__Sordariomycetes sp
OTU052	0	0	0	0	0	0	3	393	k__Fungi; p__Basidiomycota; c__Exobasidiomycetes; o__Incertae sedis; f__Incertae sedis; g__Tilletiopsis; s__Tilletiopsis pallescens
OTU053	99	33	57	170	0	3	2	7	k__Fungi; p__Ascomycota; c__Dothideomycetes; o__Pleosporales; f__unidentified; g__unidentified; s__Pleosporales sp
OTU054	38	21	91	115	15	11	21	5	k__Fungi; p__Ascomycota; c__Dothideomycetes; o__Pleosporales; f__unidentified; g__unidentified; s__Pleosporales sp
OTU055	38	67	85	92	15	8	2	2	k__Fungi; p__Ascomycota; c__Dothideomycetes; o__Pleosporales; f__Incertae sedis; g__Periconia; s__Periconia byssoides
OTU056	39	13	145	22	21	14	15	20	k__Fungi; p__Ascomycota; c__unidentified; o__unidentified; f__unidentified; g__unidentified; s__Ascomycota sp
OTU057	54	68	55	37	21	17	16	7	k__Fungi; p__Ascomycota; c__Dothideomycetes; o__Pleosporales; f__Pleosporaceae; g__unidentified; s__Pleosporaceae sp
OTU058	16	5	25	23	24	44	31	35	k__Fungi; p__Basidiomycota; c__Microbotryomycetes; o__Sporidiobolales; f__Incertae sedis; g__Rhodotorula; s__Rhodotorula marina
OTU059	32	2	228	2	0	4	7	0	k__Fungi; p__Ascomycota; c__Sordariomycetes; o__unidentified; f__unidentified; g__unidentified; s__Sordariomycetes sp
OTU060	21	3	17	7	17	124	24	17	k__Fungi; p__Ascomycota; c__Sordariomycetes; o__unidentified; f__unidentified; g__unidentified; s__Sordariomycetes sp
OTU061	190	1	35	1	0	0	0	0	k__Fungi; p__Ascomycota; c__Sordariomycetes; o__Hypocreales; f__Ophiocordycipitaceae; g__unidentified; s__Ophiocordycipitaceae sp
OTU062	2	0	7	1	36	19	1	146	k__Plantae; p__unidentified; c__unidentified; o__unidentified; f__unidentified; g__unidentified; s__Plantae sp
OTU063	15	12	75	24	6	23	19	8	k__Fungi; p__Ascomycota; c__Dothideomycetes; o__Pleosporales; f__Pleosporaceae; g__Stemphylium; s__Stemphylium herbarum
OTU064	8	19	43	35	18	10	18	15	k__Fungi; p__Basidiomycota; c__Cystobasidiomycetes; o__Erythrobasidiales; f__Incertae sedis; g__Erythrobasidium; s__Erythrobasidium hasegawianum
OTU065	41	85	23	10	4	3	3	2	k__Fungi; p__Basidiomycota; c__Tremellomycetes; o__Tremellales; f__Incertae sedis; g__Cryptococcus; s__Cryptococcus paraflavus
OTU066	34	9	13	17	6	14	37	27	k__Fungi; p__Ascomycota; c__Eurotiomycetes; o__Eurotiales; f__Trichocomaceae; g__Penicillium; s__Penicillium sp

OTU067	10	39	50	37	2	2	1	31	k__Fungi; p__Basidiomycota; c__Tremellomycetes; o__Tremellales; f__Incertae sedis; g__Cryptococcus; s__Cryptococcus victoriae
OTU068	40	10	27	20	17	7	26	24	k__Fungi; p__Ascomycota; c__Saccharomycetes; o__Saccharomycetales; f__unidentified; g__unidentified; s__Saccharomycetales sp
OTU069	3	0	2	2	42	21	34	21	k__Fungi; p__Ascomycota; c__Dothideomycetes; o__Dothideales; f__Dothioraceae; g__Aureobasidium; s__Aureobasidium pullulans
OTU070	0	4	0	0	0	135	28	0	k__Fungi; p__Basidiomycota; c__Agaricomycetes; o__Geastrales; f__Geastraceae; g__Geastrum; s__Geastrum corollinum
OTU071	6	26	81	32	2	2	1	4	k__Fungi; p__Basidiomycota; c__Tremellomycetes; o__Tremellales; f__Incertae sedis; g__Dioszegia; s__Dioszegia takashimae
OTU072	49	31	15	10	4	29	14	8	k__Fungi; p__Ascomycota; c__Archaeorhizomycetes; o__Archaeorhizomycetales; f__Archaeorhizomycetaceae; g__Archaeorhizomyces; s__Archaeorhizomyces sp
OTU073	34	9	20	26	21	11	17	15	k__Fungi; p__Ascomycota; c__Saccharomycetes; o__Saccharomycetales; f__Incertae sedis; g__Yarrowia; s__Yarrowia lipolytica
OTU074	17	4	6	2	6	8	0	2	k__Fungi; p__Basidiomycota; c__Exobasidiomycetes; o__Exobasidiales; f__unidentified; g__unidentified; s__Exobasidiales sp
OTU075	2	0	108	0	0	0	28	0	No blast hit
OTU076	8	7	35	46	6	3	3	9	k__Fungi; p__Basidiomycota; c__Agaricomycetes; o__Polyporales; f__unidentified; g__unidentified; s__Polyporales sp
OTU077	1	30	15	1	10	75	1	0	k__Fungi; p__Ascomycota; c__Dothideomycetes; o__unidentified; f__unidentified; g__unidentified; s__Dothideomycetes sp
OTU078	2	8	5	17	25	0	6	65	k__Fungi; p__Basidiomycota; c__Exobasidiomycetes; o__Incertae sedis; f__Incertae sedis; g__Acaromyces; s__Acaromyces ingoldii
OTU079	88	14	10	18	0	0	0	0	k__Fungi; p__Basidiomycota; c__Pucciniomycetes; o__Septobasidiales; f__Septobasidiaceae; g__Septobasidium; s__Septobasidium arachnoideum
OTU080	0	1	30	1	85	1	3	0	k__Fungi; p__Ascomycota; c__Sordariomycetes; o__Hypocreales; f__Incertae sedis; g__Sarocladium; s__Sarocladium sp
OTU081	0	0	0	0	0	98	22	1	k__Fungi; p__Ascomycota; c__Eurotiomycetes; o__Eurotiales; f__Trichocomaceae; g__Penicillium; s__Penicillium cvjetkovicii
OTU082	0	0	0	0	11	17	77	5	k__Fungi; p__Basidiomycota; c__Exobasidiomycetes; o__Microstromatales; f__Quambalariaceae; g__Quambalaria; s__Quambalaria coyrecup

OTU083	0	0	17	0	0	0	101	0	No blast hit
OTU084	0	3	0	0	83	0	0	32	k__Fungi; p__Ascomycota; c__Lecanoromycetes; o__Ostropales; f__Stictidaceae; g__Stictis; s__Stictis confusa
OTU085	0	0	4	4	5	4	1	8	k__Fungi; p__Basidiomycota; c__Exobasidiomycetes; o__Incertae sedis; f__Incertae sedis; g__Tilletiopsis; s__Tilletiopsis minor
OTU086	2	110	0	2	0	1	1	0	k__Fungi; p__Basidiomycota; c__Tremellomycetes; o__Tremellales; f__Incertae sedis; g__Hannaella; s__Hannaella oryzae
OTU087	8	56	5	3	6	2	21	8	k__Fungi; p__Ascomycota; c__Eurotiomycetes; o__Eurotiales; f__Trichocomaceae; g__Aspergillus; s__Aspergillus cibarius
OTU088	10	7	88	8	0	0	0	0	k__Fungi; p__Ascomycota; c__Sordariomycetes; o__Xylariales; f__Amphisphaeriaceae; g__unidentified; s__Amphisphaeriaceae sp
OTU089	7	0	3	0	55	0	27	4	k__Fungi; p__Ascomycota; c__Eurotiomycetes; o__Eurotiales; f__Trichocomaceae; g__Talaromyces; s__Talaromyces rotundus
OTU090	0	0	0	2	0	0	0	103	k__Fungi; p__Ascomycota; c__Sordariomycetes; o__Hypocreales; f__Nectriaceae; g__Nectria; s__Nectria sp
OTU091	1	0	0	0	102	0	0	1	k__Fungi; p__Basidiomycota; c__Tremellomycetes; o__Tremellales; f__Incertae sedis; g__Cryptococcus; s__Cryptococcus heimaeyensis
OTU092	17	3	29	4	12	5	24	5	k__Fungi; p__Ascomycota; c__Eurotiomycetes; o__Eurotiales; f__Thermoascaceae; g__Thermoascus; s__Thermoascus aurantiacus
OTU093	17	9	23	0	10	8	23	3	k__Fungi; p__Ascomycota; c__Sordariomycetes; o__Diaporthales; f__Togniniaceae; g__Phaeoacremonium; s__Phaeoacremonium hungaricum
OTU094	6	4	39	8	3	0	5	4	k__Fungi; p__Basidiomycota; c__Microbotryomycetes; o__Sporidiobolales; f__Incertae sedis; g__Rhodotorula; s__Rhodotorula aurantiaca
OTU095	48	0	24	2	4	0	0	14	k__Fungi; p__Basidiomycota; c__Agaricomycetes; o__Cantharellales; f__Botryobasidiaceae; g__unidentified; s__Botryobasidiaceae sp
OTU096	0	0	0	0	29	3	25	30	k__Fungi; p__Basidiomycota; c__Exobasidiomycetes; o__Microstromatales; f__Microstromataceae; g__Microstroma; s__Microstroma juglandis
OTU097	16	5	2	9	3	0	6	2	k__Fungi; p__Ascomycota; c__Sordariomycetes; o__Hypocreales; f__Nectriaceae; g__Gibberella; s__Gibberella tricincta
OTU098	14	1	20	8	5	16	11	2	k__Fungi; p__Ascomycota; c__Saccharomycetes; o__Saccharomycetales; f__Incertae sedis; g__Candida; s__Candida xylopsoci
OTU099	10	17	17	5	11	5	12	1	k__Fungi; p__Ascomycota; c__Eurotiomycetes; o__Eurotiales; f__Trichocomaceae; g__Aspergillus; s__Aspergillus flavus

OTU100	78	0	0	0	0	0	0	0	k__Fungi; p__Ascomycota; c__Dothideomycetes; o__Pleosporales; f__Incertae sedis; g__Phaeomycocentrospora; s__Phaeomycocentrospora cantuariensis
OTU101	2	9	4	1	26	14	2	15	k__Fungi; p__Basidiomycota; c__Exobasidiomycetes; o__Incertae sedis; f__Incertae sedis; g__Tilletiopsis; s__Tilletiopsis pallescens
OTU102	0	0	0	0	0	0	75	2	k__Fungi; p__unidentified; c__unidentified; o__unidentified; f__unidentified; g__unidentified; s__Fungi sp
OTU103	16	0	9	0	1	4	38	7	No blast hit
OTU104	0	73	1	3	0	0	0	0	k__Fungi; p__Ascomycota; c__Dothideomycetes; o__Capnodiales; f__Mycosphaerellaceae; g__Sphaerulina; s__Sphaerulina sp
OTU105	0	2	6	3	22	15	12	12	k__Fungi; p__Ascomycota; c__Sordariomycetes; o__Hypocreales; f__Incertae sedis; g__Acremonium; s__Acremonium nepalense
OTU106	12	3	11	12	4	0	24	1	k__Fungi; p__Ascomycota; c__Dothideomycetes; o__Pleosporales; f__Incertae sedis; g__unidentified; s__Incertae sedis sp
OTU107	8	5	57	0	0	2	0	4	No blast hit
OTU108	10	15	33	11	2	1	1	1	k__Fungi; p__Ascomycota; c__Dothideomycetes; o__Pleosporales; f__unidentified; g__unidentified; s__Pleosporales sp
OTU109	6	2	7	0	0	0	0	58	k__Fungi; p__Ascomycota; c__Saccharomycetes; o__Saccharomycetales; f__unidentified; g__unidentified; s__Saccharomycetales sp
OTU110	0	72	0	0	0	0	0	1	k__Fungi; p__Basidiomycota; c__Exobasidiomycetes; o__Microstromatales; f__Quambalariaceae; g__Quambalaria; s__Quambalaria cyanescens
OTU111	0	0	13	8	12	2	26	3	k__Fungi; p__Ascomycota; c__Dothideomycetes; o__Pleosporales; f__Sporormiaceae; g__unidentified; s__Sporormiaceae sp
OTU112	3	2	2	0	7	7	30	11	k__Fungi; p__Ascomycota; c__Eurotiomycetes; o__Eurotiales; f__Trichocomaceae; g__Aspergillus; s__Aspergillus penicillioides
OTU113	7	1	20	6	11	4	1	2	k__Fungi; p__Basidiomycota; c__Tremellomycetes; o__Tremellales; f__Incertae sedis; g__Hannaella; s__Hannaella siamensis
OTU114	5	0	51	2	0	10	0	0	k__Fungi; p__Ascomycota; c__Sordariomycetes; o__Hypocreales; f__Incertae sedis; g__Sarocladium; s__Sarocladium glaucum
OTU115	0	1	2	0	14	2	9	35	k__Fungi; p__Basidiomycota; c__Exobasidiomycetes; o__unidentified; f__unidentified; g__unidentified; s__Exobasidiomycetes sp
OTU116	1	2	0	5	30	5	1	0	k__Fungi; p__Ascomycota; c__Sordariomycetes; o__Incertae sedis; f__Plectosphaerellaceae; g__Lectera; s__Lectera longa

OTU117	8	0	0	0	0	3	55	0	k__Fungi; p__Basidiomycota; c__Agaricomycetes; o__Phallales; f__Phallaceae; g__Phallus; s__Phallus hadriani
OTU118	48	0	0	1	0	0	4	3	k__Fungi; p__Ascomycota; c__unidentified; o__unidentified; f__unidentified; g__unidentified; s__Ascomycota sp
OTU119	6	52	3	1	2	0	0	0	k__Fungi; p__Ascomycota; c__unidentified; o__unidentified; f__unidentified; g__unidentified; s__Ascomycota sp
OTU120	0	33	0	2	2	18	7	0	k__Fungi; p__Ascomycota; c__Eurotiomycetes; o__Eurotiales; f__Trichocomaceae; g__Aspergillus; s__Aspergillus ochraceus
OTU121	0	0	1	0	0	61	0	0	k__Fungi; p__Ascomycota; c__Sordariomycetes; o__Hypocreales; f__Incertae sedis; g__Acremonium; s__Acremonium sp
OTU122	0	3	43	5	2	3	3	0	k__Fungi; p__Ascomycota; c__Sordariomycetes; o__Hypocreales; f__Cordycipitaceae; g__Cordyceps; s__Cordyceps bassiana
OTU123	4	16	7	26	1	0	0	2	k__Fungi; p__Ascomycota; c__Dothideomycetes; o__Pleosporales; f__unidentified; g__unidentified; s__Pleosporales sp
OTU124	6	16	14	11	2	0	0	3	k__Plantae; p__unidentified; c__unidentified; o__unidentified; f__unidentified; g__unidentified; s__Plantae sp
OTU125	0	0	3	0	38	7	0	11	k__Fungi; p__Basidiomycota; c__Tremellomycetes; o__Tremellales; f__Incertae sedis; g__Cryptococcus; s__Cryptococcus heimaeyensis
OTU126	1	0	0	0	0	0	1	1	k__Fungi; p__Ascomycota; c__Sordariomycetes; o__Xylariales; f__unidentified; g__unidentified; s__Xylariales sp
OTU127	22	14	11	1	3	6	0	1	k__Fungi; p__Ascomycota; c__Archaeorhizomycetes; o__Archaeorhizomycetales; f__Archaeorhizomycetaceae; g__Archaeorhizomyces; s__Archaeorhizomyces sp
OTU128	0	5	7	0	13	21	1	9	k__Fungi; p__Ascomycota; c__Eurotiomycetes; o__Incertae sedis; f__Monascaceae; g__Monascus; s__Monascus purpureus
OTU129	8	3	20	2	8	5	4	2	k__Fungi; p__Basidiomycota; c__Agaricomycetes; o__Agaricales; f__Schizophyllaceae; g__Schizophyllum; s__Schizophyllum commune
OTU130	0	0	0	2	1	16	32	1	k__Fungi; p__Basidiomycota; c__Agaricomycetes; o__Phallales; f__Phallaceae; g__Phallus; s__Phallus rugulosus
OTU131	3	11	27	0	3	5	4	0	k__Fungi; p__Ascomycota; c__Sordariomycetes; o__Hypocreales; f__Incertae sedis; g__Sarocladium; s__Sarocladium sp
OTU132	9	2	1	1	25	2	8	5	k__Fungi; p__Ascomycota; c__Sordariomycetes; o__Hypocreales; f__Cordycipitaceae; g__Simplicillium; s__Simplicillium aogashimaense
OTU133	1	4	7	7	3	4	20	4	k__Fungi; p__Basidiomycota; c__Wallemiomycetes; o__Wallemiales; f__Wallemiaceae; g__Wallemia; s__Wallemia sebi

OTU134	0	0	0	0	29	1	22	0	k__Fungi; p__Ascomycota; c__Eurotiomycetes; o__Eurotiales; f__Trichocomaceae; g__Penicillium; s__Penicillium virgatum
OTU135	17	10	4	2	3	4	2	3	k__Fungi; p__Ascomycota; c__Leotiomycetes; o__Erysiphales; f__Erysiphaceae; g__Podosphaera; s__Podosphaera gunnerae
OTU136	13	20	15	3	0	0	0	0	k__Fungi; p__Basidiomycota; c__Agaricomycetes; o__unidentified; f__unidentified; g__unidentified; s__Agaricomycetes sp
OTU137	0	3	6	5	34	0	0	1	k__Fungi; p__Ascomycota; c__Sordariomycetes; o__Hypocreales; f__Bionectriaceae; g__unidentified; s__Bionectriaceae sp
OTU138	17	2	6	2	8	2	9	1	k__Fungi; p__Ascomycota; c__Sordariomycetes; o__Hypocreales; f__Cordycipitaceae; g__unidentified; s__Cordycipitaceae sp
OTU139	0	1	2	0	9	1	0	33	k__Plantae; p__unidentified; c__unidentified; o__unidentified; f__unidentified; g__unidentified; s__Plantae sp
OTU140	0	2	10	0	9	19	6	1	k__Fungi; p__Ascomycota; c__unidentified; o__unidentified; f__unidentified; g__unidentified; s__Ascomycota sp
OTU141	8	10	6	2	1	9	5	1	k__Fungi; p__Ascomycota; c__Incertae sedis; o__Incertae sedis; f__Incertae sedis; g__Torula; s__Torula caligans
OTU142	0	0	0	0	5	5	26	7	k__Fungi; p__Ascomycota; c__Eurotiomycetes; o__Eurotiales; f__Trichocomaceae; g__Aspergillus; s__Aspergillus penicillioides
OTU143	0	32	3	3	0	0	5	1	k__Fungi; p__Ascomycota; c__Dothideomycetes; o__unidentified; f__unidentified; g__unidentified; s__Dothideomycetes sp
OTU144	0	0	1	0	1	21	18	2	k__Fungi; p__Ascomycota; c__unidentified; o__unidentified; f__unidentified; g__unidentified; s__Ascomycota sp
OTU145	0	2	0	0	1	3	5	34	k__Fungi; p__Basidiomycota; c__Tremellomycetes; o__Tremellales; f__Incertae sedis; g__Cryptococcus; s__Cryptococcus sp
OTU146	0	0	1	0	0	39	0	5	k__Fungi; p__Ascomycota; c__Sordariomycetes; o__Hypocreales; f__Ophiocordycipitaceae; g__Purpureocillium; s__Purpureocillium lilacinum
OTU147	5	0	8	16	1	1	0	5	k__Fungi; p__Ascomycota; c__Dothideomycetes; o__Capnodiales; f__Mycosphaerellaceae; g__Stenella; s__Stenella araguata
OTU148	1	3	2	1	0	6	1	29	k__Fungi; p__Ascomycota; c__Sordariomycetes; o__Hypocreales; f__Incertae sedis; g__Acremonium; s__Acremonium persicinum
OTU149	17	15	5	0	0	7	0	0	k__Fungi; p__Basidiomycota; c__Tremellomycetes; o__Tremellales; f__Incertae sedis; g__Dioszegia; s__Dioszegia sp
OTU150	1	4	21	11	1	0	1	0	k__Fungi; p__Ascomycota; c__Dothideomycetes; o__Pleosporales; f__Phaeosphaeriaceae; g__unidentified; s__Phaeosphaeriaceae sp

OTU151	3	7	13	19	0	0	0	0	k__Fungi; p__Ascomycota; c__Dothideomycetes; o__Pleosporales; f__Leptosphaeriaceae; g__Coniothyrium; s__Coniothyrium sidae
OTU152	15	10	5	11	0	0	0	0	k__Fungi; p__Ascomycota; c__unidentified; o__unidentified; f__unidentified; g__unidentified; s__Ascomycota sp
OTU153	0	9	16	5	0	0	0	0	k__Fungi; p__Ascomycota; c__Dothideomycetes; o__unidentified; f__unidentified; g__unidentified; s__Dothideomycetes sp
OTU154	24	8	6	2	0	0	0	0	k__Fungi; p__Basidiomycota; c__Tremellomycetes; o__Tremellales; f__unidentified; g__unidentified; s__Tremellales sp
OTU155	0	0	0	0	29	5	0	5	No blast hit
OTU156	0	15	0	6	2	2	6	5	k__Fungi; p__Basidiomycota; c__Tremellomycetes; o__Tremellales; f__Incertae sedis; g__Cryptococcus; s__Cryptococcus rajasthanensis
OTU157	0	2	5	3	1	1	14	8	k__Fungi; p__Ascomycota; c__Dothideomycetes; o__Capnodiales; f__Mycosphaerellaceae; g__Mycosphaerella; s__Mycosphaerella tassiana
OTU158	2	2	3	16	0	1	4	0	k__Fungi; p__Ascomycota; c__Dothideomycetes; o__Dothideales; f__Dothioraceae; g__Aureobasidium; s__Aureobasidium microstictum
OTU159	3	2	14	3	0	4	9	1	k__Fungi; p__Basidiomycota; c__Agaricomycetes; o__Agaricales; f__unidentified; g__unidentified; s__Agaricales sp
OTU160	2	8	3	18	0	0	3	3	k__Fungi; p__Ascomycota; c__unidentified; o__unidentified; f__unidentified; g__unidentified; s__Ascomycota sp
OTU161	5	7	6	7	2	3	2	2	k__Fungi; p__Ascomycota; c__Dothideomycetes; o__unidentified; f__unidentified; g__unidentified; s__Dothideomycetes sp
OTU162	0	0	0	0	1	34	0	0	k__Fungi; p__Basidiomycota; c__Exobasidiomycetes; o__Microstromatales; f__Incertae sedis; g__Jaminaea; s__Jaminaea sp
OTU163	11	1	6	3	3	1	10	0	k__Fungi; p__Basidiomycota; c__Incertae sedis; o__Malasseziales; f__Malasseziaceae; g__Malassezia; s__Malassezia restricta
OTU164	0	0	0	0	0	0	0	32	k__Fungi; p__Ascomycota; c__Incertae sedis; o__Incertae sedis; f__Incertae sedis; g__Hansfordia; s__Hansfordia pulvinata
OTU165	0	1	11	8	0	0	0	4	k__Fungi; p__Basidiomycota; c__Microbotryomycetes; o__Sporidiobolales; f__Incertae sedis; g__Sporobolomyces; s__Sporobolomyces oryzicola
OTU166	0	24	0	1	5	0	5	0	k__Fungi; p__Ascomycota; c__Eurotiomycetes; o__Eurotiales; f__Trichocomaceae; g__Penicillium; s__Penicillium citrinum
OTU167	0	0	0	0	0	0	0	35	k__Fungi; p__Basidiomycota; c__Exobasidiomycetes; o__Exobasidiales; f__Exobasidiaceae; g__Exobasidium; s__Exobasidium gracile

OTU168	0	3	1	1	2	1	0	26	k__Fungi; p__Basidiomycota; c__Tremellomycetes; o__Tremellales; f__Incertae sedis; g__Cryptococcus; s__Cryptococcus sp
OTU169	0	0	0	0	28	0	0	4	k__Fungi; p__Basidiomycota; c__Ustilaginomycetes; o__Ustilaginales; f__Ustilaginaceae; g__unidentified; s__Ustilaginaceae sp
OTU170	2	5	6	8	4	2	4	3	k__Fungi; p__Ascomycota; c__Sordariomycetes; o__Hypocreales; f__Nectriaceae; g__Fusarium; s__Fusarium keratoplasticum
OTU171	10	7	8	8	0	0	0	0	k__Fungi; p__Ascomycota; c__Dothideomycetes; o__Capnodiales; f__Mycosphaerellaceae; g__unidentified; s__Mycosphaerellaceae sp
OTU172	0	0	0	2	2	5	4	6	k__Fungi; p__Ascomycota; c__Dothideomycetes; o__Dothideales; f__Dothioraceae; g__Aureobasidium; s__Aureobasidium pullulans
OTU173	4	2	26	0	0	0	0	0	k__Fungi; p__Basidiomycota; c__Agaricomycetes; o__Agaricales; f__unidentified; g__unidentified; s__Agaricales sp
OTU174	2	23	2	2	0	0	0	1	k__Fungi; p__Ascomycota; c__Sordariomycetes; o__Hypocreales; f__unidentified; g__unidentified; s__Hypocreales sp
OTU175	3	2	4	7	0	0	2	2	k__Fungi; p__Ascomycota; c__Dothideomycetes; o__Pleosporales; f__Incertae sedis; g__Phoma; s__Phoma calidophila
OTU176	12	2	8	3	5	1	0	0	k__Plantae; p__unidentified; c__unidentified; o__unidentified; f__unidentified; g__unidentified; s__Plantae sp
OTU177	0	1	1	0	11	2	2	1	k__Fungi; p__Ascomycota; c__Dothideomycetes; o__Pleosporales; f__Pleosporaceae; g__unidentified; s__Pleosporaceae sp
OTU178	0	0	3	7	3	1	3	9	k__Fungi; p__Ascomycota; c__Leotiomycetes; o__Helotiales; f__Sclerotiniaceae; g__Botrytis; s__Botrytis caroliniana
OTU179	0	0	0	0	6	4	7	7	k__Fungi; p__Ascomycota; c__Dothideomycetes; o__Dothideales; f__Dothioraceae; g__Aureobasidium; s__Aureobasidium pullulans
OTU180	3	0	10	5	8	1	0	0	k__Fungi; p__Ascomycota; c__Sordariomycetes; o__Sordariales; f__Chaetomiaceae; g__unidentified; s__Chaetomiaceae sp
OTU181	8	6	0	0	2	11	0	0	k__Fungi; p__Ascomycota; c__unidentified; o__unidentified; f__unidentified; g__unidentified; s__Ascomycota sp
OTU182	0	6	10	0	8	0	0	0	k__Fungi; p__Basidiomycota; c__Tremellomycetes; o__Tremellales; f__Incertae sedis; g__Cryptococcus; s__Cryptococcus flavus
OTU183	2	4	21	1	0	0	0	0	k__Fungi; p__Basidiomycota; c__Agaricomycetes; o__Agaricales; f__unidentified; g__unidentified; s__Agaricales sp
OTU184	25	0	0	2	0	0	0	0	k__Fungi; p__Basidiomycota; c__Tremellomycetes; o__Tremellales; f__Incertae sedis; g__Fellomyces; s__Fellomyces sichuanensis

OTU185	9	4	3	5	0	0	5	1	k__Fungi; p__Ascomycota; c__Sordariomycetes; o__Hypocreales; f__Incertae sedis; g__Acremonium; s__Acremonium dichromosporum
OTU186	26	0	0	0	0	0	0	0	k__Fungi; p__Basidiomycota; c__Agaricomycetes; o__Hymenochaetales; f__Schizoporaceae; g__Xylodon; s__Xylodon erastii
OTU187	1	15	5	1	2	0	2	0	k__Fungi; p__Ascomycota; c__Dothideomycetes; o__Pleosporales; f__unidentified; g__unidentified; s__Pleosporales sp
OTU188	2	0	0	1	0	16	0	6	k__Fungi; p__Ascomycota; c__Eurotiomycetes; o__Eurotiales; f__Trichocomaceae; g__Penicillium; s__Penicillium melinii
OTU189	1	2	4	5	2	2	1	0	k__Fungi; p__Ascomycota; c__unidentified; o__unidentified; f__unidentified; g__unidentified; s__Ascomycota sp
OTU190	0	0	0	0	0	0	25	0	k__Fungi; p__Ascomycota; c__Sordariomycetes; o__Hypocreales; f__Hypocreaceae; g__Hypocrea; s__Hypocrea lactea
OTU191	4	1	6	1	0	3	1	0	k__Fungi; p__Ascomycota; c__Eurotiomycetes; o__Eurotiales; f__Trichocomaceae; g__Aspergillus; s__Aspergillus piperis
OTU192	1	4	8	4	3	2	0	0	k__Fungi; p__Ascomycota; c__Dothideomycetes; o__Pleosporales; f__Pleosporaceae; g__Bipolaris; s__Bipolaris maydis
OTU193	0	0	3	4	0	0	0	2	k__Fungi; p__Basidiomycota; c__Tremellomycetes; o__Tremellales; f__Incertae sedis; g__Bullera; s__Bullera pseudoalba
OTU194	0	0	0	0	1	1	6	4	No blast hit
OTU195	0	2	2	15	3	0	0	0	k__Fungi; p__Ascomycota; c__Dothideomycetes; o__Capnodiales; f__Teratosphaeriaceae; g__Devriesia; s__Devriesia pseudoamericana
OTU196	6	2	11	2	0	0	0	0	k__Fungi; p__Ascomycota; c__Dothideomycetes; o__Pleosporales; f__Incertae sedis; g__Periconia; s__Periconia byssoides
OTU197	0	1	6	8	1	1	0	5	k__Fungi; p__Basidiomycota; c__unidentified; o__unidentified; f__unidentified; g__unidentified; s__Basidiomycota sp
OTU198	0	0	0	3	0	1	2	16	k__Fungi; p__Ascomycota; c__Saccharomycetes; o__Saccharomycetales; f__Pichiaceae; g__Pichia; s__Pichia kluyveri
OTU199	0	0	22	0	0	0	0	0	k__Fungi; p__Ascomycota; c__Leotiomycetes; o__Erysiphales; f__Erysiphaceae; g__Blumeria; s__Blumeria graminis
OTU200	3	3	4	9	0	0	0	2	k__Fungi; p__Ascomycota; c__Leotiomycetes; o__Helotiales; f__unidentified; g__unidentified; s__Helotiales sp
OTU201	0	0	0	3	6	0	9	2	k__Fungi; p__Ascomycota; c__Dothideomycetes; o__Pleosporales; f__Montagnulaceae; g__Paraphaeosphaeria; s__Paraphaeosphaeria barriae

OTU202	1	0	6	0	9	2	2	1	k__Plantae; p__unidentified; c__unidentified; o__unidentified; f__unidentified; g__unidentified; s__Plantae sp
OTU203	2	0	6	0	2	1	7	4	No blast hit
OTU204	0	0	0	0	3	18	0	0	k__Fungi; p__Ascomycota; c__Eurotiomycetes; o__Eurotiales; f__Trichocomaceae; g__unidentified; s__Trichocomaceae sp
OTU205	0	1	21	0	0	0	0	0	k__Fungi; p__Ascomycota; c__Dothideomycetes; o__Capnodiales; f__Incertae sedis; g__Rachicladosporium; s__Rachicladosporium cboliae
OTU206	2	4	2	0	3	4	3	3	k__Fungi; p__Ascomycota; c__Saccharomycetes; o__Saccharomycetales; f__Incertae sedis; g__Candida; s__Candida sake
OTU207	0	5	9	0	0	5	0	2	k__Fungi; p__Ascomycota; c__Sordariomycetes; o__Hypocreales; f__Hypocreaceae; g__unidentified; s__Hypocreaceae sp
OTU208	0	0	0	0	15	0	6	0	k__Fungi; p__Ascomycota; c__Eurotiomycetes; o__Eurotiales; f__Trichocomaceae; g__Penicillium; s__Penicillium sp
OTU209	0	0	0	0	4	2	1	9	k__Fungi; p__Basidiomycota; c__Exobasidiomycetes; o__Incertae sedis; f__Incertae sedis; g__Tilletiopsis; s__Tilletiopsis pallescens
OTU210	19	0	0	0	2	0	0	0	k__Fungi; p__Ascomycota; c__Archaeorhizomycetes; o__Archaeorhizomycetales; f__Archaeorhizomycetaceae; g__Archaeorhizomyces; s__Archaeorhizomyces sp
OTU211	0	0	2	17	0	0	1	1	k__Fungi; p__Ascomycota; c__Dothideomycetes; o__Pleosporales; f__unidentified; g__unidentified; s__Pleosporales sp
OTU212	0	0	0	12	6	0	3	0	k__Fungi; p__Ascomycota; c__Sordariomycetes; o__Hypocreales; f__Incertae sedis; g__Acremonium; s__Acremonium sp
OTU213	14	0	0	0	6	0	0	0	k__Fungi; p__Basidiomycota; c__Tremellomycetes; o__Tremellales; f__Incertae sedis; g__Bullera; s__Bullera sp
OTU214	0	0	0	0	0	0	1	5	k__Fungi; p__Basidiomycota; c__Exobasidiomycetes; o__Incertae sedis; f__Incertae sedis; g__Tilletiopsis; s__Tilletiopsis pallescens
OTU215	0	2	0	0	4	2	3	5	k__Fungi; p__Ascomycota; c__Leotiomycetes; o__Erysiphales; f__Erysiphaceae; g__Podosphaera; s__Podosphaera leucotricha
OTU216	1	0	1	3	1	0	8	3	k__Fungi; p__Basidiomycota; c__Tremellomycetes; o__Cystofilobasidiales; f__Cystofilobasidiaceae; g__Guehomyces; s__Guehomyces pullulans
OTU217	0	0	0	0	0	19	0	0	k__Fungi; p__Ascomycota; c__Eurotiomycetes; o__Eurotiales; f__Trichocomaceae; g__Penicillium; s__Penicillium sclerotiorum
OTU218	0	0	0	0	10	9	0	0	k__Plantae; p__unidentified; c__unidentified; o__unidentified; f__unidentified; g__unidentified; s__Plantae sp

OTU219	5	3	1	4	0	1	0	1	k__Fungi; p__Ascomycota; c__unidentified; o__unidentified; f__unidentified; g__unidentified; s__Ascomycota sp
OTU220	0	0	16	0	1	0	0	1	k__Fungi; p__unidentified; c__unidentified; o__unidentified; f__unidentified; g__unidentified; s__Fungi sp
OTU221	0	0	18	0	0	0	1	0	No blast hit
OTU222	1	1	3	3	3	0	6	2	No blast hit
OTU223	0	0	2	0	0	17	0	0	k__Fungi; p__Ascomycota; c__Sordariomycetes; o__Sordariales; f__Lasiosphaeriaceae; g__Apodospora; s__Apodospora peruviana
OTU224	1	0	8	0	1	2	4	0	k__Fungi; p__Basidiomycota; c__Agaricomycetes; o__Auriculariales; f__Incertae sedis; g__Auricularia; s__Auricularia delicata
OTU225	0	0	0	0	16	2	0	0	k__Fungi; p__Ascomycota; c__Eurotiomycetes; o__Eurotiales; f__Trichocomaceae; g__Penicillium; s__Penicillium anatolicum
OTU226	0	1	0	0	2	3	5	6	k__Fungi; p__Ascomycota; c__Sordariomycetes; o__Hypocreales; f__Incertae sedis; g__Myrothecium; s__Myrothecium verrucaria
OTU227	0	0	0	0	0	3	2	13	No blast hit
OTU228	4	0	1	7	0	0	3	0	k__Fungi; p__unidentified; c__unidentified; o__unidentified; f__unidentified; g__unidentified; s__Fungi sp
OTU229	0	1	0	0	2	7	2	6	k__Fungi; p__Ascomycota; c__Sordariomycetes; o__Hypocreales; f__Incertae sedis; g__Acremonium; s__Acremonium sp
OTU230	2	0	6	4	0	3	1	0	k__Fungi; p__Ascomycota; c__Dothideomycetes; o__Pleosporales; f__Pleosporaceae; g__Chalastospora; s__Chalastospora ellipsoidea
OTU231	1	0	6	2	2	1	4	0	k__Fungi; p__Ascomycota; c__Eurotiomycetes; o__Eurotiales; f__Trichocomaceae; g__Talaromyces; s__Talaromyces marneffei
OTU232	1	0	4	3	1	0	2	3	k__Fungi; p__Basidiomycota; c__Agaricomycetes; o__unidentified; f__unidentified; g__unidentified; s__Agaricomycetes sp
OTU233	0	0	0	1	8	2	0	5	k__Fungi; p__Basidiomycota; c__Microbotryomycetes; o__Sporidiobolales; f__Incertae sedis; g__Sporobolomyces; s__Sporobolomyces ruberrimus
OTU234	0	0	1	2	3	7	0	3	k__Fungi; p__Basidiomycota; c__Microbotryomycetes; o__Sporidiobolales; f__unidentified; g__unidentified; s__Sporidiobolales sp
OTU235	0	0	5	4	1	0	0	7	k__Fungi; p__Chytridiomycota; c__Chytridiomycetes; o__Spizellomycetales; f__Spizellomycetaceae; g__Triparticalcar; s__Triparticalcar sp

OTU236	0	4	0	3	3	0	0	0	k__Fungi; p__Ascomycota; c__Dothideomycetes; o__Capnodiales; f__Mycosphaerellaceae; g__Mycosphaerella; s__Mycosphaerella berkeleyi
OTU237	1	14	0	1	0	0	0	0	k__Fungi; p__Ascomycota; c__Dothideomycetes; o__Capnodiales; f__Mycosphaerellaceae; g__Dissoconium; s__Dissoconium proteae
OTU238	0	1	6	10	0	0	0	0	k__Fungi; p__unidentified; c__unidentified; o__unidentified; f__unidentified; g__unidentified; s__Fungi sp
OTU239	4	1	4	1	0	2	3	0	k__Fungi; p__Ascomycota; c__unidentified; o__unidentified; f__unidentified; g__unidentified; s__Ascomycota sp
OTU240	0	2	3	2	0	0	0	0	k__Fungi; p__Basidiomycota; c__Microbotryomycetes; o__Sporidiobolales; f__Incertae sedis; g__unidentified; s__Incertae sedis sp
OTU241	0	0	16	0	0	0	0	0	k__Fungi; p__Ascomycota; c__Incertae sedis; o__Incertae sedis; f__Incertae sedis; g__Radulidium; s__Radulidium subulatum
OTU242	0	0	0	0	0	0	0	0	k__Fungi; p__Ascomycota; c__Sordariomycetes; o__Hypocreales; f__Incertae sedis; g__Acremonium; s__Acremonium alternatum
OTU243	0	8	4	3	0	0	1	0	k__Fungi; p__Ascomycota; c__Leotiomycetes; o__Erysiphales; f__Erysiphaceae; g__Phyllactinia; s__Phyllactinia pyri-serotinae
OTU244	9	0	6	1	0	0	0	0	k__Fungi; p__Basidiomycota; c__Tremellomycetes; o__Tremellales; f__Incertae sedis; g__Bullera; s__Bullera globospora
OTU245	8	0	0	0	5	0	0	0	k__Fungi; p__Ascomycota; c__Saccharomycetes; o__Saccharomycetales; f__Incertae sedis; g__Candida; s__Candida apicola
OTU246	3	4	2	0	0	3	3	1	k__Fungi; p__Ascomycota; c__Eurotiomycetes; o__Eurotiales; f__Trichocomaceae; g__Penicillium; s__Penicillium pimiteouiense
OTU247	0	0	0	0	2	5	1	8	k__Fungi; p__Ascomycota; c__Sordariomycetes; o__Hypocreales; f__Incertae sedis; g__Acremonium; s__Acremonium sp
OTU248	0	0	0	14	0	0	0	0	k__Fungi; p__Ascomycota; c__Dothideomycetes; o__Pleosporales; f__Incertae sedis; g__Phoma; s__Phoma calidophila
OTU249	3	3	6	1	0	0	0	0	k__Fungi; p__Basidiomycota; c__Ustilaginomycetes; o__Ustilaginales; f__Ustilaginaceae; g__unidentified; s__Ustilaginaceae sp
OTU250	0	0	15	0	0	0	0	0	k__Fungi; p__Ascomycota; c__Dothideomycetes; o__Pleosporales; f__unidentified; g__unidentified; s__Pleosporales sp
OTU251	2	4	3	5	0	0	0	0	k__Fungi; p__Ascomycota; c__Dothideomycetes; o__Pleosporales; f__Phaeosphaeriaceae; g__Sclerostagonospora; s__Sclerostagonospora opuntiae
OTU252	0	1	1	0	0	2	0	11	k__Plantae; p__unidentified; c__unidentified; o__unidentified; f__unidentified; g__unidentified; s__Plantae sp

OTU253	0	15	0	0	0	0	0	0	k__Fungi; p__Ascomycota; c__Dothideomycetes; o__Capnodiales; f__Mycosphaerellaceae; g__Mycosphaerella; s__Mycosphaerella pseudomarksii
OTU254	0	0	1	0	2	3	4	3	k__Fungi; p__Ascomycota; c__Dothideomycetes; o__Dothideales; f__Dothioraceae; g__Aureobasidium; s__Aureobasidium pullulans
OTU255	0	0	1	1	2	9	0	2	k__Fungi; p__Ascomycota; c__Sordariomycetes; o__unidentified; f__unidentified; g__unidentified; s__Sordariomycetes sp
OTU256	2	2	7	3	0	0	0	0	k__Fungi; p__Ascomycota; c__Dothideomycetes; o__unidentified; f__unidentified; g__unidentified; s__Dothideomycetes sp
OTU257	1	6	3	5	0	0	0	0	k__Fungi; p__Ascomycota; c__Leotiomycetes; o__Helotiales; f__Helotiaceae; g__Articulospora; s__Articulospora sp
OTU258	0	0	13	1	0	0	0	0	No blast hit
OTU259	1	0	0	0	0	3	10	0	k__Fungi; p__Ascomycota; c__Eurotiomycetes; o__Eurotiales; f__Trichocomaceae; g__Penicillium; s__Penicillium anatolicum
OTU260	3	1	2	2	0	0	4	0	k__Fungi; p__Ascomycota; c__Eurotiomycetes; o__Eurotiales; f__unidentified; g__unidentified; s__Eurotiales sp
OTU261	12	0	0	0	0	1	0	0	k__Fungi; p__Ascomycota; c__Sordariomycetes; o__Hypocreales; f__Incertae sedis; g__Stachybotrys; s__Stachybotrys sp
OTU262	2	1	10	0	0	1	0	0	k__Fungi; p__Ascomycota; c__Eurotiomycetes; o__Eurotiales; f__Incertae sedis; g__Thermomyces; s__Thermomyces lanuginosus
OTU263	0	1	1	5	3	3	1	0	k__Fungi; p__Ascomycota; c__unidentified; o__unidentified; f__unidentified; g__unidentified; s__Ascomycota sp
OTU264	0	0	0	0	3	2	7	1	k__Fungi; p__Ascomycota; c__Eurotiomycetes; o__Eurotiales; f__Trichocomaceae; g__Penicillium; s__Penicillium polonicum
OTU265	0	0	0	0	0	0	1	12	k__Fungi; p__Basidiomycota; c__Exobasidiomycetes; o__Exobasidiales; f__Exobasidiaceae; g__Exobasidium; s__Exobasidium miyabei
OTU266	0	0	14	0	0	0	0	0	k__Fungi; p__Basidiomycota; c__Tremellomycetes; o__Tremellales; f__Incertae sedis; g__unidentified; s__Incertae sedis sp
OTU267	0	5	3	0	2	0	1	2	k__Fungi; p__Ascomycota; c__Dothideomycetes; o__Venturiales; f__Venturiaceae; g__Venturia; s__Venturia asperata
OTU268	0	0	0	0	0	0	14	0	k__Fungi; p__Ascomycota; c__Eurotiomycetes; o__Ascosphaerales; f__Ascosphaeraceae; g__Ascosphaera; s__Ascosphaera apis
OTU269	0	9	3	2	0	0	0	0	k__Fungi; p__Ascomycota; c__Dothideomycetes; o__Pleosporales; f__Incertae sedis; g__Didymella; s__Didymella exigua

OTU270	9	5	0	0	0	0	0	0	k__Fungi; p__Basidiomycota; c__Agaricomycetes; o__Trechisporales; f__Hydnodontaceae; g__Trechispora; s__Trechispora sp
OTU271	0	3	3	7	0	0	0	0	k__Fungi; p__Basidiomycota; c__Pucciniomycetes; o__Septobasidiales; f__Septobasidiaceae; g__Septobasidium; s__Septobasidium sp
OTU272	0	1	0	0	1	4	0	7	k__Fungi; p__Ascomycota; c__unidentified; o__unidentified; f__unidentified; g__unidentified; s__Ascomycota sp
OTU273	0	0	0	0	1	0	0	11	k__Fungi; p__Basidiomycota; c__Tremellomycetes; o__Tremellales; f__Incertae sedis; g__Cryptococcus; s__Cryptococcus heimaeyensis
OTU274	0	0	0	13	0	0	0	0	k__Fungi; p__Ascomycota; c__unidentified; o__unidentified; f__unidentified; g__unidentified; s__Ascomycota sp
OTU275	0	0	13	0	0	0	0	0	k__Fungi; p__Ascomycota; c__Dothideomycetes; o__Capnodiales; f__Mycosphaerellaceae; g__Ramichloridium; s__Ramichloridium luteum
OTU276	0	0	0	13	0	0	0	0	k__Fungi; p__unidentified; c__unidentified; o__unidentified; f__unidentified; g__unidentified; s__Fungi sp
OTU277	0	0	3	0	7	0	3	0	k__Fungi; p__Ascomycota; c__Saccharomycetes; o__Saccharomycetales; f__Pichiaceae; g__Pichia; s__Pichia fermentans
OTU278	1	0	3	3	0	0	0	1	k__Fungi; p__Ascomycota; c__Eurotiomycetes; o__Eurotiales; f__Trichocomaceae; g__Aspergillus; s__Aspergillus japonicus
OTU279	0	0	3	1	2	1	0	3	k__Fungi; p__Basidiomycota; c__Tremellomycetes; o__Tremellales; f__Incertae sedis; g__Cryptococcus; s__Cryptococcus albidus
OTU280	12	0	0	0	0	0	0	0	k__Fungi; p__Basidiomycota; c__Tremellomycetes; o__Tremellales; f__Incertae sedis; g__Cryptococcus; s__Cryptococcus sp
OTU281	0	4	1	2	3	0	2	0	k__Fungi; p__Ascomycota; c__Sordariomycetes; o__Sordariales; f__unidentified; g__unidentified; s__Sordariales sp
OTU282	0	3	2	0	1	1	4	0	k__Fungi; p__Rozellomycota; c__unidentified; o__unidentified; f__unidentified; g__unidentified; s__Rozellomycota sp
OTU283	0	0	0	0	0	0	0	12	k__Fungi; p__Basidiomycota; c__Tremellomycetes; o__Tremellales; f__Incertae sedis; g__Bullera; s__Bullera sp
OTU284	0	0	0	0	0	0	12	0	k__Fungi; p__Ascomycota; c__Sordariomycetes; o__Hypocreales; f__Incertae sedis; g__Acremonium; s__Acremonium sp
OTU285	0	0	10	0	2	0	0	0	k__Fungi; p__Ascomycota; c__Sordariomycetes; o__Microascales; f__Microascaceae; g__Scedosporium; s__Scedosporium minutisporum
OTU286	0	2	0	0	1	1	6	0	k__Fungi; p__Ascomycota; c__Eurotiomycetes; o__Eurotiales; f__Trichocomaceae; g__Aspergillus; s__Aspergillus penicillioides

OTU287	2	0	2	0	1	1	3	1	k__Fungi; p__Basidiomycota; c__Tremellomycetes; o__Tremellales; f__Incertae sedis; g__Cryptococcus; s__Cryptococcus cyanovorans
OTU288	1	3	0	2	0	0	0	2	k__Fungi; p__Ascomycota; c__Dothideomycetes; o__Pleosporales; f__unidentified; g__unidentified; s__Pleosporales sp
OTU289	0	0	0	0	0	11	0	0	k__Fungi; p__Ascomycota; c__Dothideomycetes; o__unidentified; f__unidentified; g__unidentified; s__Dothideomycetes sp
OTU290	11	0	0	0	0	0	0	0	k__Fungi; p__Basidiomycota; c__Tremellomycetes; o__Tremellales; f__Incertae sedis; g__Cryptococcus; s__Cryptococcus sp
OTU291	5	0	1	2	1	0	1	0	k__Fungi; p__Ascomycota; c__Eurotiomycetes; o__Eurotiales; f__Trichocomaceae; g__Penicillium; s__Penicillium levitum
OTU292	8	1	0	0	0	0	2	0	k__Fungi; p__Ascomycota; c__Saccharomycetes; o__Saccharomycetales; f__Incertae sedis; g__Candida; s__Candida catenulata
OTU293	0	1	4	3	0	0	0	1	k__Fungi; p__Ascomycota; c__Dothideomycetes; o__Pleosporales; f__Lophiostomataceae; g__Lophiostoma; s__Lophiostoma macrostomum
OTU294	0	0	0	0	0	11	0	0	k__Fungi; p__Ascomycota; c__Eurotiomycetes; o__Eurotiales; f__Trichocomaceae; g__Penicillium; s__Penicillium coffeae
OTU295	7	0	0	0	1	2	0	1	k__Fungi; p__Basidiomycota; c__Tremellomycetes; o__Tremellales; f__Incertae sedis; g__Cryptococcus; s__Cryptococcus podzolicus
OTU296	0	0	0	11	0	0	0	0	k__Fungi; p__Ascomycota; c__Sordariomycetes; o__Sordariales; f__Lasiosphaeriaceae; g__Arnium; s__Arnium gigantosporum
OTU297	2	1	0	6	0	0	1	0	k__Fungi; p__Ascomycota; c__unidentified; o__unidentified; f__unidentified; g__unidentified; s__Ascomycota sp
OTU298	8	1	0	2	0	0	0	0	k__Fungi; p__Basidiomycota; c__Tremellomycetes; o__Tremellales; f__Incertae sedis; g__Tremella; s__Tremella sp
OTU299	0	1	6	3	0	0	0	0	k__Fungi; p__Ascomycota; c__Incertae sedis; o__Incertae sedis; f__Incertae sedis; g__Knufia; s__Knufia epidermidis
OTU300	0	0	10	0	0	0	0	0	k__Fungi; p__Ascomycota; c__Sordariomycetes; o__Hypocreales; f__Cordycipitaceae; g__Isaria; s__Isaria javanica
OTU301	5	2	0	0	0	1	1	0	k__Fungi; p__Basidiomycota; c__Tremellomycetes; o__Trichosporonales; f__Trichosporonaceae; g__Trichosporon; s__Trichosporon cutaneum
OTU302	0	1	3	1	4	0	0	0	k__Fungi; p__Ascomycota; c__Dothideomycetes; o__unidentified; f__unidentified; g__unidentified; s__Dothideomycetes sp
OTU303	0	10	0	0	0	0	0	0	k__Fungi; p__Ascomycota; c__Sordariomycetes; o__Hypocreales; f__Ophiocordycipitaceae; g__Hirsutella; s__Hirsutella vermicola

OTU304	3	0	4	1	0	0	0	1	k__Fungi; p__Basidiomycota; c__Cystobasidiomycetes; o__Cystobasidiales; f__Cystobasidiaceae; g__Occultifur; s__Occultifur externus
OTU305	0	0	10	0	0	0	0	0	k__Fungi; p__Ascomycota; c__Lecanoromycetes; o__Peltigerales; f__Collemataceae; g__Leptogium; s__Leptogium saturninum
OTU306	0	10	0	0	0	0	0	0	k__Plantae; p__unidentified; c__unidentified; o__unidentified; f__unidentified; g__unidentified; s__Plantae sp
OTU307	0	0	10	0	0	0	0	0	k__Fungi; p__Ascomycota; c__Incertae sedis; o__Incertae sedis; f__Incertae sedis; g__Radulidium; s__Radulidium subulatum
OTU308	2	0	1	2	1	0	2	2	k__Fungi; p__Ascomycota; c__Sordariomycetes; o__Hypocreales; f__Cordycipitaceae; g__Engyodontium; s__Engyodontium album
OTU309	0	1	1	5	0	0	2	0	k__Fungi; p__Basidiomycota; c__Ustilaginomycetes; o__Ustilaginales; f__Ustilaginaceae; g__Ustilago; s__Ustilago drakensbergiana
OTU310	1	0	3	4	0	0	0	0	k__Fungi; p__Basidiomycota; c__Ustilaginomycetes; o__Ustilaginales; f__Ustilaginaceae; g__Ustilago; s__Ustilago maydis
OTU311	0	0	3	4	0	0	0	2	k__Fungi; p__Basidiomycota; c__Agaricomycetes; o__Polyporales; f__Polyporaceae; g__Trametes; s__Trametes hirsuta
OTU312	7	1	0	0	0	2	0	0	k__Fungi; p__Basidiomycota; c__Agaricomycetes; o__Geastrales; f__Geastraceae; g__Geastrum; s__Geastrum lageniforme
OTU313	1	1	2	5	0	0	1	0	k__Fungi; p__Ascomycota; c__Dothideomycetes; o__Pleosporales; f__Incertae sedis; g__Phoma; s__Phoma calidophila
OTU314	0	0	6	0	0	3	0	0	k__Fungi; p__Basidiomycota; c__Agaricomycetes; o__Agaricales; f__Amanitaceae; g__Amanita; s__Amanita crocea
OTU315	9	0	0	0	0	0	0	0	k__Plantae; p__unidentified; c__unidentified; o__unidentified; f__unidentified; g__unidentified; s__Plantae sp
OTU316	0	3	3	3	0	0	0	0	k__Fungi; p__Ascomycota; c__Dothideomycetes; o__Myriangiales; f__unidentified; g__unidentified; s__Myriangiales sp
OTU317	0	0	0	0	0	9	0	0	k__Fungi; p__Ascomycota; c__Eurotiomycetes; o__Eurotiales; f__Trichocomaceae; g__Aspergillus; s__Aspergillus caesiellus
OTU318	1	0	1	0	0	1	0	4	k__Fungi; p__Ascomycota; c__Eurotiomycetes; o__Incertae sedis; f__Incertae sedis; g__Sarcinomyces; s__Sarcinomyces sp
OTU319	5	2	0	0	1	1	0	0	k__Fungi; p__Ascomycota; c__Archaeorhizomycetes; o__Archaeorhizomycetales; f__Archaeorhizomycetaceae; g__Archaeorhizomyces; s__Archaeorhizomyces sp
OTU320	0	0	0	3	0	1	5	0	k__Fungi; p__Ascomycota; c__Dothideomycetes; o__Pleosporales; f__Pleosporaceae; g__Exserohilum; s__Exserohilum rostratum

OTU321	5	0	0	0	0	0	0	4	k__Fungi; p__Ascomycota; c__Dothideomycetes; o__Pleosporales; f__Lophiostomataceae; g__Lophiostoma; s__Lophiostoma cynaroidis
OTU322	0	3	0	0	0	0	0	0	k__Fungi; p__Basidiomycota; c__Tremellomycetes; o__Tremellales; f__Incertae sedis; g__Auriculibuller; s__Auriculibuller fuscus
OTU323	5	0	4	0	0	0	0	0	k__Fungi; p__Ascomycota; c__Sordariomycetes; o__Melanosporales; f__Ceratostomataceae; g__Harzia; s__Harzia acremonioides
OTU324	0	0	0	0	2	0	5	1	k__Fungi; p__unidentified; c__unidentified; o__unidentified; f__unidentified; g__unidentified; s__Fungi sp
OTU325	0	0	0	0	0	0	8	0	k__Fungi; p__Basidiomycota; c__Tremellomycetes; o__Tremellales; f__Incertae sedis; g__Bullera; s__Bullera sp
OTU326	0	0	0	0	8	0	0	0	k__Fungi; p__Ascomycota; c__Sordariomycetes; o__unidentified; f__unidentified; g__unidentified; s__Sordariomycetes sp
OTU327	0	0	6	1	0	0	0	0	k__Protista; p__Ciliophora; c__unidentified; o__unidentified; f__unidentified; g__unidentified; s__Ciliophora sp
OTU328	0	2	0	3	0	1	0	0	k__Fungi; p__Basidiomycota; c__Tremellomycetes; o__Tremellales; f__Incertae sedis; g__Hannaella; s__Hannaella sinensis
OTU329	0	5	0	0	0	3	0	0	k__Fungi; p__Ascomycota; c__Sordariomycetes; o__Hypocreales; f__Incertae sedis; g__Acremonium; s__Acremonium tubakii
OTU330	0	0	1	0	2	1	4	0	k__Fungi; p__Ascomycota; c__Saccharomycetes; o__Saccharomycetales; f__Incertae sedis; g__Candida; s__Candida blattae
OTU331	0	0	5	0	0	0	3	0	k__Fungi; p__Basidiomycota; c__Agaricomycetes; o__Cantharellales; f__Botryobasidiaceae; g__unidentified; s__Botryobasidiaceae sp
OTU332	0	3	3	0	0	0	0	2	k__Fungi; p__Ascomycota; c__Dothideomycetes; o__Pleosporales; f__Incertae sedis; g__Phoma; s__Phoma paspali
OTU333	0	0	0	1	5	0	0	1	k__Plantae; p__unidentified; c__unidentified; o__unidentified; f__unidentified; g__unidentified; s__Plantae sp
OTU334	3	0	4	0	0	0	0	0	k__Fungi; p__Basidiomycota; c__Tremellomycetes; o__Tremellales; f__Incertae sedis; g__unidentified; s__Incertae sedis sp
OTU335	0	0	7	0	0	0	0	0	k__Fungi; p__Ascomycota; c__Dothideomycetes; o__Pleosporales; f__Sporormiaceae; g__unidentified; s__Sporormiaceae sp
OTU336	8	0	0	0	0	0	0	0	k__Fungi; p__Ascomycota; c__Dothideomycetes; o__Pleosporales; f__Incertae sedis; g__unidentified; s__Incertae sedis sp
OTU337	0	0	0	0	0	0	0	8	k__Fungi; p__Basidiomycota; c__Tremellomycetes; o__Tremellales; f__Incertae sedis; g__Bullera; s__Bullera sp

OTU338	2	0	3	0	0	0	3	0	k__Fungi; p__Ascomycota; c__Dothideomycetes; o__unidentified; f__unidentified; g__unidentified; s__Dothideomycetes sp
OTU339	0	0	0	0	5	0	0	1	k__Fungi; p__Ascomycota; c__Leotiomycetes; o__Helotiales; f__Dermateaceae; g__unidentified; s__Dermateaceae sp
OTU340	0	0	0	0	1	2	2	1	k__Fungi; p__Ascomycota; c__Dothideomycetes; o__Dothideales; f__Dothioraceae; g__Aureobasidium; s__Aureobasidium pullulans
OTU341	5	0	3	0	0	0	0	0	k__Fungi; p__unidentified; c__unidentified; o__unidentified; f__unidentified; g__unidentified; s__Fungi sp
OTU342	0	2	3	2	0	0	0	0	k__Fungi; p__Ascomycota; c__Dothideomycetes; o__Pleosporales; f__unidentified; g__unidentified; s__Pleosporales sp
OTU343	0	0	0	0	4	0	2	1	k__Fungi; p__Ascomycota; c__Eurotiomycetes; o__Ascosphaerales; f__Ascosphaeraceae; g__Ascosphaera; s__Ascosphaera proliperda
OTU344	0	0	7	0	0	0	0	0	k__Fungi; p__Ascomycota; c__Dothideomycetes; o__Pleosporales; f__Pleosporaceae; g__unidentified; s__Pleosporaceae sp
OTU345	0	1	0	0	6	0	0	0	k__Fungi; p__Ascomycota; c__unidentified; o__unidentified; f__unidentified; g__unidentified; s__Ascomycota sp
OTU346	2	0	2	3	0	0	0	0	No blast hit
OTU347	0	0	1	2	0	0	4	0	k__Fungi; p__Basidiomycota; c__Tremellomycetes; o__Tremellales; f__Incertae sedis; g__Bullera; s__Bullera globospora
OTU348	0	0	6	0	0	0	0	1	k__Fungi; p__Ascomycota; c__Archaeorhizomycetes; o__Archaeorhizomycetales; f__Archaeorhizomycetaceae; g__Archaeorhizomyces; s__Archaeorhizomyces sp
OTU349	0	0	5	0	1	1	0	0	k__Fungi; p__Ascomycota; c__Dothideomycetes; o__Pleosporales; f__unidentified; g__unidentified; s__Pleosporales sp
OTU350	0	0	7	0	0	0	0	0	k__Fungi; p__unidentified; c__unidentified; o__unidentified; f__unidentified; g__unidentified; s__Fungi sp
OTU351	0	7	0	0	0	0	0	0	k__Fungi; p__Ascomycota; c__Sordariomycetes; o__Incertae sedis; f__Incertae sedis; g__Myrmecridium; s__Myrmecridium flexuosum
OTU352	0	0	0	0	0	0	0	2	k__Fungi; p__Ascomycota; c__Dothideomycetes; o__Venturiales; f__Venturiaceae; g__Venturia; s__Venturia nashicola
OTU353	0	6	1	0	0	0	0	0	k__Fungi; p__Ascomycota; c__Sordariomycetes; o__Hypocreales; f__Cordycipitaceae; g__Lecanicillium; s__Lecanicillium fungicola

OTU354	7	0	0	0	0	0	0	0	k__Fungi; p__Ascomycota; c__Incertae sedis; o__Incertae sedis; f__Incertae sedis; g__Phaeocytostroma; s__Phaeocytostroma ambiguum
OTU355	1	1	2	1	0	0	0	0	k__Fungi; p__Ascomycota; c__Sordariomycetes; o__Xylariales; f__unidentified; g__unidentified; s__Xylariales sp
OTU356	7	0	0	0	0	0	0	0	k__Fungi; p__Ascomycota; c__Archaeorhizomycetes; o__Archaeorhizomycetales; f__Archaeorhizomycetaceae; g__Archaeorhizomyces; s__Archaeorhizomyces sp
OTU357	0	1	1	2	0	0	0	0	k__Fungi; p__Ascomycota; c__Dothideomycetes; o__Dothideales; f__Dothioraceae; g__Aureobasidium; s__Aureobasidium microstictum
OTU358	0	0	0	0	0	3	0	0	k__Fungi; p__Ascomycota; c__Dothideomycetes; o__Pleosporales; f__unidentified; g__unidentified; s__Pleosporales sp
OTU359	0	1	0	0	2	0	0	2	k__Fungi; p__Basidiomycota; c__Agaricomycetes; o__Agaricales; f__Psathyrellaceae; g__Coprinopsis; s__Coprinopsis calospora
OTU360	0	7	0	0	0	0	0	0	k__Plantae; p__unidentified; c__unidentified; o__unidentified; f__unidentified; g__unidentified; s__Plantae sp
OTU361	0	2	1	0	4	0	0	0	k__Fungi; p__Ascomycota; c__Sordariomycetes; o__Hypocreales; f__Incertae sedis; g__Acremonium; s__Acremonium alcalophilum
OTU362	0	0	0	0	0	0	1	5	k__Fungi; p__Ascomycota; c__Sordariomycetes; o__Hypocreales; f__Incertae sedis; g__Acremonium; s__Acremonium alternatum
OTU363	1	2	0	0	4	0	0	0	k__Fungi; p__Ascomycota; c__Dothideomycetes; o__Pleosporales; f__Pleosporaceae; g__unidentified; s__Pleosporaceae sp
OTU364	0	0	2	0	1	4	0	0	k__Fungi; p__Ascomycota; c__Dothideomycetes; o__Capnodiales; f__Incertae sedis; g__Capnobotryella; s__Capnobotryella sp
OTU365	0	0	3	0	1	0	0	3	k__Fungi; p__Basidiomycota; c__Agaricomycetes; o__Agaricales; f__Psathyrellaceae; g__Coprinopsis; s__Coprinopsis clastophylla
OTU366	1	2	4	0	0	0	0	0	k__Fungi; p__Ascomycota; c__Lecanoromycetes; o__Peltigerales; f__Pannariaceae; g__unidentified; s__Pannariaceae sp
OTU367	0	0	0	0	0	6	1	0	k__Plantae; p__unidentified; c__unidentified; o__unidentified; f__unidentified; g__unidentified; s__Plantae sp
OTU368	1	1	0	1	0	0	3	0	k__Fungi; p__Ascomycota; c__Sordariomycetes; o__Hypocreales; f__Hypocreaceae; g__Trichoderma; s__Trichoderma atroviride
OTU369	2	1	0	0	2	0	1	1	No blast hit

OTU370	7	0	0	0	0	0	0	0	k__Fungi; p__Basidiomycota; c__Agaricomycetes; o__Boletales; f__Boletaceae; g__unidentified; s__Boletaceae sp
OTU371	0	1	2	1	0	0	0	2	k__Fungi; p__Ascomycota; c__Dothideomycetes; o__Pleosporales; f__unidentified; g__unidentified; s__Pleosporales sp
OTU372	3	0	2	1	1	0	0	0	k__Fungi; p__Ascomycota; c__Lecanoromycetes; o__Teloschistales; f__Teloschistaceae; g__Austroplaca; s__Austroplaca millegrana
OTU373	0	0	0	1	0	0	6	0	k__Fungi; p__Ascomycota; c__Dothideomycetes; o__Dothideales; f__Dothioraceae; g__Aureobasidium; s__Aureobasidium pullulans
OTU374	1	1	1	1	1	0	1	0	k__Fungi; p__Ascomycota; c__unidentified; o__unidentified; f__unidentified; g__unidentified; s__Ascomycota sp
OTU375	0	5	0	0	1	0	0	0	k__Fungi; p__Ascomycota; c__Sordariomycetes; o__Incertae sedis; f__Incertae sedis; g__Savoryella; s__Savoryella appendiculata
OTU376	6	0	0	0	0	0	0	0	k__Fungi; p__Basidiomycota; c__Microbotryomycetes; o__Sporidiobolales; f__Incertae sedis; g__Sporobolomyces; s__Sporobolomyces poonsookiae
OTU377	0	0	0	0	6	0	0	0	k__Fungi; p__Basidiomycota; c__Wallemiomycetes; o__Geminibasidiales; f__Geminibasidiaceae; g__Geminibasidium; s__Geminibasidium sp
OTU378	2	0	0	3	0	0	0	0	k__Fungi; p__Basidiomycota; c__Microbotryomycetes; o__Sporidiobolales; f__Incertae sedis; g__Sporobolomyces; s__Sporobolomyces sp
OTU379	0	6	0	0	0	0	0	0	k__Fungi; p__Chytridiomycota; c__unidentified; o__unidentified; f__unidentified; g__unidentified; s__Chytridiomycota sp
OTU380	1	1	1	0	0	0	2	0	k__Fungi; p__Ascomycota; c__Eurotiomycetes; o__Eurotiales; f__Trichocomaceae; g__Aspergillus; s__Aspergillus penicillioides
OTU381	0	0	0	0	0	0	6	0	k__Fungi; p__unidentified; c__unidentified; o__unidentified; f__unidentified; g__unidentified; s__Fungi sp
OTU382	0	0	2	2	1	0	1	0	k__Fungi; p__Ascomycota; c__Lecanoromycetes; o__Peltigerales; f__Lobariaceae; g__Lobaria; s__Lobaria kurokawae
OTU383	0	0	0	2	1	0	0	0	k__Fungi; p__Ascomycota; c__Sordariomycetes; o__Hypocreales; f__unidentified; g__unidentified; s__Hypocreales sp
OTU384	0	0	0	0	4	0	0	0	k__Fungi; p__Ascomycota; c__Sordariomycetes; o__Xylariales; f__Xylariaceae; g__Coniolariella; s__Coniolariella hispanica
OTU385	0	0	0	0	0	0	0	0	k__Plantae; p__unidentified; c__unidentified; o__unidentified; f__unidentified; g__unidentified; s__Plantae sp
OTU386	0	0	0	0	1	2	0	3	k__Plantae; p__unidentified; c__unidentified; o__unidentified; f__unidentified; g__unidentified; s__Plantae sp

OTU387	0	0	0	0	0	0	0	4	k__Fungi; p__Ascomycota; c__Dothideomycetes; o__Dothideales; f__Dothioraceae; g__Aureobasidium; s__Aureobasidium pullulans
OTU388	1	0	3	0	0	2	0	0	k__Fungi; p__Ascomycota; c__Sordariomycetes; o__Hypocreales; f__Incertae sedis; g__Stachybotrys; s__Stachybotrys chartarum
OTU389	0	1	0	2	0	0	0	2	k__Fungi; p__Basidiomycota; c__Agaricomycetes; o__Agaricales; f__Psathyrellaceae; g__Psathyrella; s__Psathyrella candolleana
OTU390	1	1	0	3	0	0	0	1	k__Fungi; p__Ascomycota; c__Sordariomycetes; o__Magnaporthales; f__Magnaporthaceae; g__unidentified; s__Magnaporthaceae sp
OTU391	0	1	4	0	0	0	0	0	k__Fungi; p__Ascomycota; c__Sordariomycetes; o__unidentified; f__unidentified; g__unidentified; s__Sordariomycetes sp
OTU392	0	0	1	1	3	0	0	1	k__Fungi; p__Ascomycota; c__Leotiomycetes; o__Helotiales; f__Dermateaceae; g__unidentified; s__Dermateaceae sp
OTU393	1	1	1	1	0	1	0	0	k__Fungi; p__Ascomycota; c__Sordariomycetes; o__Xylariales; f__unidentified; g__unidentified; s__Xylariales sp
OTU394	0	0	0	0	2	0	4	0	No blast hit
OTU395	0	0	0	1	0	1	0	1	k__Fungi; p__Ascomycota; c__Dothideomycetes; o__Pleosporales; f__Sporormiaceae; g__Sporormiella; s__Sporormiella australis
OTU396	0	0	0	0	0	6	0	0	k__Fungi; p__Ascomycota; c__Dothideomycetes; o__Pleosporales; f__unidentified; g__unidentified; s__Pleosporales sp
OTU397	0	1	5	0	0	0	0	0	k__Fungi; p__Ascomycota; c__Archaeorhizomycetes; o__Archaeorhizomycetales; f__Archaeorhizomycetaceae; g__Archaeorhizomyces; s__Archaeorhizomyces borealis
OTU398	0	0	0	1	0	2	0	1	k__Fungi; p__Basidiomycota; c__Tremellomycetes; o__Tremellales; f__Incertae sedis; g__Cryptococcus; s__Cryptococcus sp
OTU399	0	0	0	0	0	0	6	0	No blast hit
OTU400	0	5	0	0	1	0	0	0	k__Fungi; p__Ascomycota; c__Eurotiomycetes; o__Eurotiales; f__Thermoascaceae; g__unidentified; s__Thermoascaceae sp
OTU401	0	1	0	0	0	1	1	3	k__Fungi; p__Ascomycota; c__Sordariomycetes; o__Hypocreales; f__Incertae sedis; g__Acremonium; s__Acremonium sp
OTU402	6	0	0	0	0	0	0	0	k__Fungi; p__Basidiomycota; c__Agaricomycetes; o__Boletales; f__Boletaceae; g__unidentified; s__Boletaceae sp
OTU403	0	0	0	0	1	0	0	2	k__Fungi; p__Ascomycota; c__Dothideomycetes; o__unidentified; f__unidentified; g__unidentified; s__Dothideomycetes sp

OTU404	0	0	6	0	0	0	0	0	No blast hit
OTU405	0	3	0	0	0	3	0	0	k__Fungi; p__Ascomycota; c__Archaeorhizomycetes; o__Archaeorhizomycetales; f__Archaeorhizomycetaceae; g__Archaeorhizomyces; s__Archaeorhizomyces sp
OTU406	0	0	0	0	1	2	2	1	No blast hit
OTU407	0	6	0	0	0	0	0	0	k__Fungi; p__Ascomycota; c__Sordariomycetes; o__Incertae sedis; f__Incertae sedis; g__Myrmecridium; s__Myrmecridium thailandicum
OTU408	0	0	6	0	0	0	0	0	k__Fungi; p__Ascomycota; c__Sordariomycetes; o__Hypocreales; f__Ophiocordycipitaceae; g__unidentified; s__Ophiocordycipitaceae sp
OTU409	0	0	0	0	5	0	1	0	k__Fungi; p__Ascomycota; c__Sordariomycetes; o__Microascales; f__Microascaceae; g__Microascus; s__Microascus brevicaulis
OTU410	2	1	0	1	0	0	0	1	k__Fungi; p__Basidiomycota; c__Microbotryomycetes; o__Sporidiobolales; f__unidentified; g__unidentified; s__Sporidiobolales sp
OTU411	0	1	0	0	1	0	1	1	k__Fungi; p__Ascomycota; c__Dothideomycetes; o__Pleosporales; f__Cucurbitariaceae; g__Pyrenochaetopsis; s__Pyrenochaetopsis leptospora
OTU412	0	0	1	0	0	0	0	2	k__Fungi; p__Ascomycota; c__Sordariomycetes; o__Hypocreales; f__unidentified; g__unidentified; s__Hypocreales sp
OTU413	0	2	0	0	0	0	1	1	k__Fungi; p__Ascomycota; c__Sordariomycetes; o__Sordariales; f__Lasiosphaeriaceae; g__unidentified; s__Lasiosphaeriaceae sp
OTU414	0	0	0	0	0	6	0	0	k__Fungi; p__Ascomycota; c__Sordariomycetes; o__Hypocreales; f__Cordycipitaceae; g__Simplicillium; s__Simplicillium obclavatum
OTU415	5	0	0	1	0	0	0	0	No blast hit
OTU416	1	0	3	2	0	0	0	0	k__Fungi; p__Basidiomycota; c__Tremellomycetes; o__Tremellales; f__Incertae sedis; g__Bullera; s__Bullera globospora
OTU417	0	0	4	0	0	1	0	0	k__Fungi; p__Ascomycota; c__unidentified; o__unidentified; f__unidentified; g__unidentified; s__Ascomycota sp
OTU418	1	0	0	0	0	0	0	3	k__Fungi; p__Ascomycota; c__Saccharomycetes; o__Saccharomycetales; f__Metschnikowiaceae; g__Metschnikowia; s__Metschnikowia pulcherrima
OTU419	0	0	0	0	0	0	0	6	k__Fungi; p__Ascomycota; c__Sordariomycetes; o__Hypocreales; f__Bionectriaceae; g__unidentified; s__Bionectriaceae sp
OTU420	5	0	1	0	0	0	0	0	k__Fungi; p__Ascomycota; c__Dothideomycetes; o__Capnodiales; f__Teratosphaeriaceae; g__Devriesia; s__Devriesia shelburniensis
OTU421	0	0	0	0	5	0	0	0	k__Fungi; p__unidentified; c__unidentified; o__unidentified; f__unidentified; g__unidentified; s__Fungi sp

Figure S1. Shared OTUs among samples identified by different groupings using a Venn diagram. (a) Shared OTUs between rural and peri-urban/suburb samples. (b) Shared OTUs among 8 separate samples.

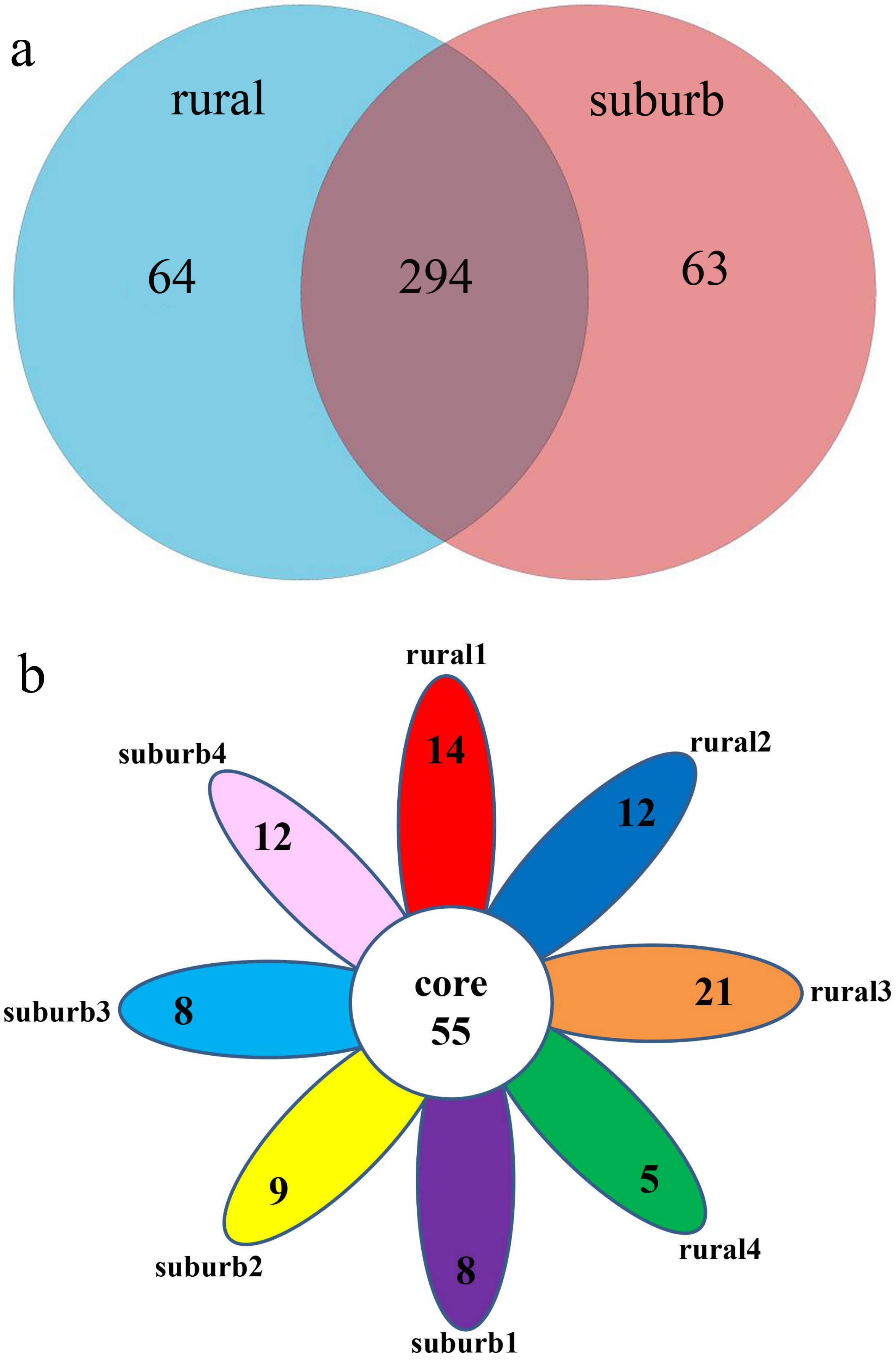

Figure S2. (a) Rarefaction curves of samples. (b) Rank abundance curves of samples.

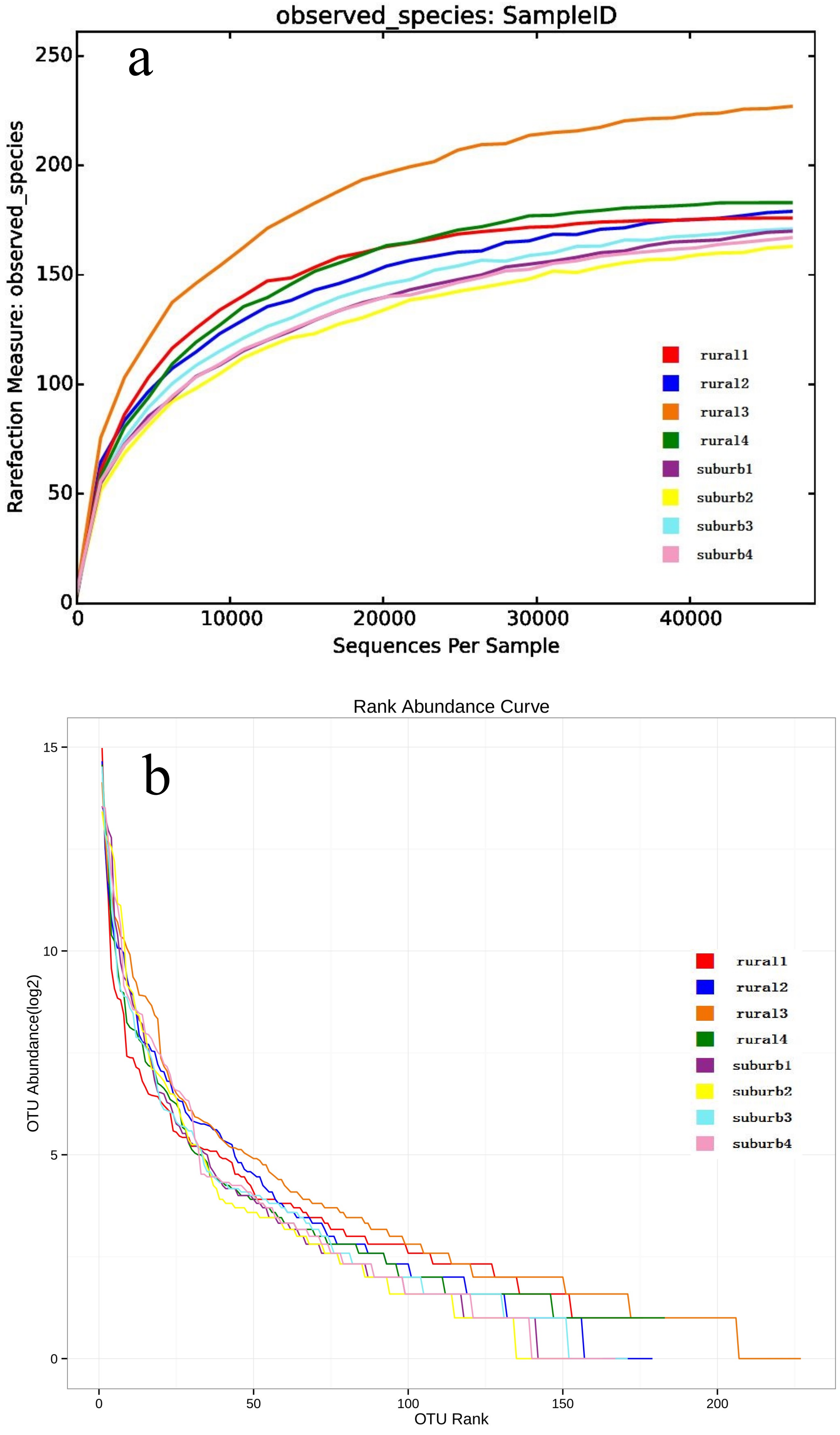

Figure S3. ACE and simpson indexes of rural and peri-urban samples. The ACE indexes of rural samples were significant higher than peri-urban samples (one-tail Student's t-test, $p<0.05$ *).

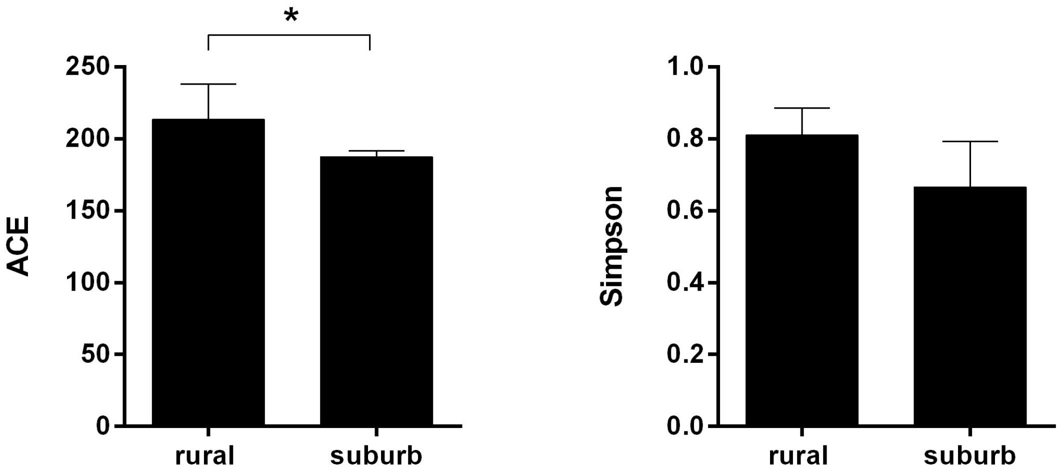

Figure S4. Classification tree showing fungal taxonomy compositions and abundance; generated by MEGAN (version 5.0).

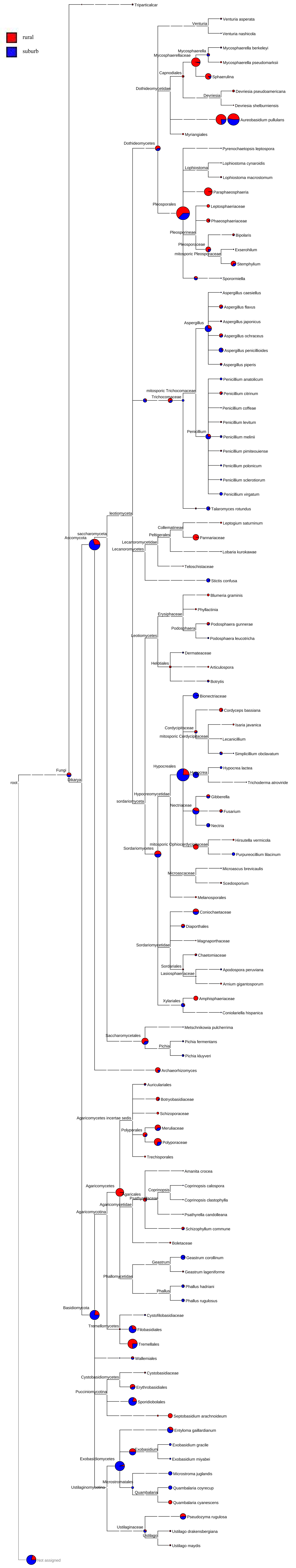

Figure S5. Taxonomy tree visualized by GraPhlAn. The background colour of each letter (A-T) is consistent with the colour of the corresponding node. The coloured nodes from the inner to outer circles represent the 20 most abundant taxa from the phylum to genus level, which are signified by letters arranged from outer to inner circles. The fan area size represents the average relative abundance of the respective taxa.

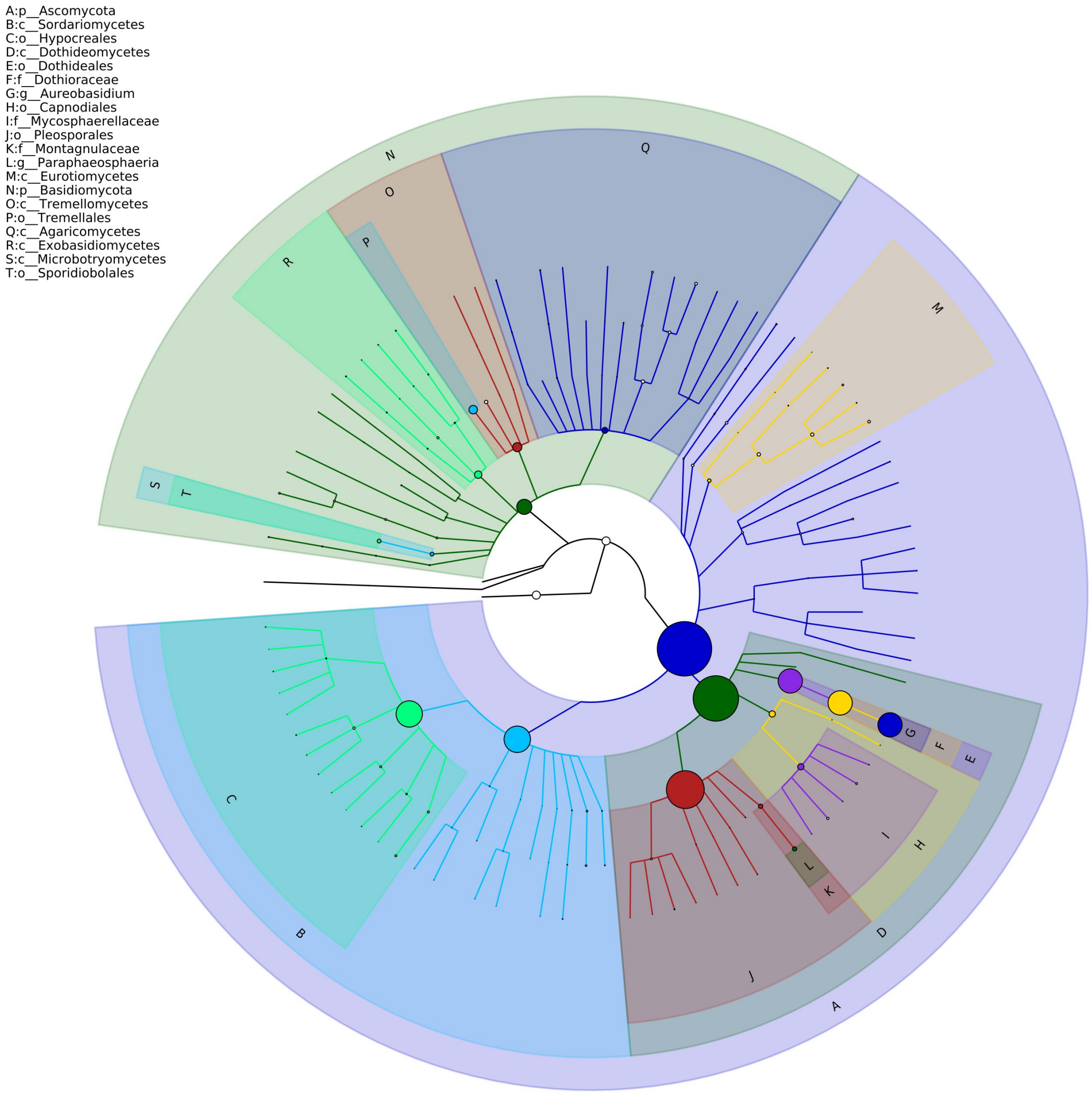

Supplement: Supplementary file 1 — Supplementary File [file 41598_2017_17436_MOESM1_ESM.pdf]
